# The Effects of Different Quantities and Qualities of Protein Intake in People with Diabetes Mellitus

**DOI:** 10.3390/nu12020365

**Published:** 2020-01-30

**Authors:** Andreas F.H. Pfeiffer, Eva Pedersen, Ursula Schwab, Ulf Risérus, Anne-Marie Aas, Matti Uusitupa, Anastasia Thanopoulou, Cyril Kendall, John L. Sievenpiper, Hana Kahleová, Dario Rahélic, Jordi Salas-Salvadó, Stephanie Gebauer, Kjeld Hermansen

**Affiliations:** 1 German Institute of Human Nutrition Potsdam-Rehbrücke, Clinical Nutrition-DZD, Arthur-Scheunert-Allee 114-116, D-14558 Nuthetal, Germany; stephanie.gebauer@gmx.de; 2 Charité University Medicine, Department of Endocrinology, Diabetes and Nutrition, Campus Benjamin Franklin, Hindenburgdamm 30, D-12203 Berlin, Germany; 3 German Center for Diabetes Research (DZD), 85764 München-Neuherberg, Germany; 4 School of Pharmacy and Medical sciences, University of South Australia, Adelaide SA 5000, Australia; eva.pedersen@ispm.unibe; 5 Institute of Public Health and Clinical Nutrition, School of Medicine, University of Eastern Finland, Kuopio Campus, 70211 Kuopio, Finland; ursula.schwab@uef.fi (U.S.); matti.uusitupa@uef.fi (M.U.); 6 Department of Medicine, Endocrinology and Clinical Nutrition, Kuopio University Hospital, 70029 KYS Kuopio, Finland; 7 Department of Public Health and Caring Sciences, Clinical Nutrition and Metabolism, Uppsala University, Box 564, 75122 Uppsala, Sweden; ulf.riserus@pubcare.uu.se; 8 Oslo University Hospital, Division of Medicine, Department of Clinical Service, Section of Nutrition and Dietetics, 0424 Oslo, Norway; a.m.aas@medisin.uio.no; 9 Diabetes Center, Second Department of Internal Medicine, Medical School, National and Kapodistian University of Athens 11527, Greece; a_thanopoulou@hotmail.com; 10 Department of Nutritional Sciences, Faculty of Medicine, University of Toronto, University of Toronto, Toronto, ON M5S 1A8, Canada; cyril.kendall@utoronto.ca (C.K.); john.sievenpiper@medportal.ca (J.L.S.); 11 Toronto 3D Knowledge Synthesis and Clinical trials Unit, Clinical Nutrition and Risk Factor Modification Centre, St. Michael’s Hospital, Toronto, ON M5C 2T2, Canada; 12 Division of Endocrinology & Metabolism, Department of Medicine, St. Michael’s Hospital, Toronto, ON M5C 2T2, Canada; 13 Li Ka Shing Knowledge Institute, St. Michael’s Hospital, Toronto, ON M5C 2T2, Canada; 14 Institute for Clinical and Experimental Medicine, 14021 Prague, Czech Republic; hana.kahleova@gmail.com; 15 Physicians Committee for Responsible Medicine, Washington, DC 20016, USA; 16 Endocrinology and Metabolic Diseases, Vuk Vrhovac University Clinic for Diabetes, Merkur University Hospital, 10000 Zagreb, Croatia; dario.rahelic@gmail.com; 17 University of Zagreb School of Medicine, 10000 Zagreb, Croatia; 18 Human Nutrition Unit, Faculty of Medicine and Health Sciences, Institute of Health Pere Virgili, Universitat Rovira i Virgili, 43201 Reus, Spain; jordi.salas@urv.cat; 19 CIBER Fisiopatología de la Obesidad y Nutrición (CIBEROBN), Instituto de Salud Carlos III, 28029 Madrid, Spain; 20 Department of Endocrinology and Metabolism, Aarhus University Hospital, 8200 AarhusAarhus, Denmark; kjeld.hermansen@aarhus.rm.dk

**Keywords:** protein intake, type 2 diabetes, hypocaloric diet, weight loss, animal protein, plant protein

## Abstract

The recommended amount and quality of protein in diets of diabetic patients are highly controversial. In order to provide evidence-based information, the Diabetes Nutrition Study Group (DNSG) used a grading procedure used for quality of evidence and strength of recommendations (GRADE). A protein intake of 10% to 20% of energy intake (E%) or about 0.8 to 1.3 g/kg body weight in people below 65 years of age, and 15% to 20% of E% in people above 65 years of age appeared safe in weight-stable conditions. There were no intervention studies addressing metabolic effects, mortality, or cardiovascular events over prolonged periods. Body weight is closely linked to metabolic control and high protein diets are often recommended. Weight-loss diets that include 23% to 32% of E% as protein for up to one year reduced blood pressure and body weight slightly but significantly more than lower protein diets, whereas blood lipids, fasting blood glucose, and HbA1c improved similarly with higher or lower protein intakes in participants with a glomerular filtration rate (GFR) >60 mL/min/1.73 m^2^. Patients with a GFR <60 mL/min/1.73 m^2^ did not show a faster decline of GFR or kidney function with protein intakes around 0.8 g/kg body weight as compared with lower intakes, thereby arguing against a restriction. The effects of protein intake on diabetic eye or nerve disease have not been reported. There are a number of studies that have compared different types of animal proteins (milk, chicken, beef, pork, and fish) or compared animal with plant protein in diabetic patients and have reported a greater reduction of serum cholesterol with plant protein. In summary, the suggested range of protein intake appears to be safe and can be adapted according to personal dietary preferences.

## 1. Introduction

Weight loss is recommended for obese people with type 2 diabetes mellitus (T2D) because obesity is closely linked to insulin resistance, elevated blood glucose, dyslipoproteinemia, and hypertension, and their associated complications. High protein diets are strongly advocated by some diabetes experts as a strategy to ease weight loss as compared with other strategies of energy restriction [1,2,3].

Surprisingly, neither the earlier nor the recently published diabetes nutrition guidelines in Great Britain [4], the USA [5], and Canada [6] address the role of protein in detail, leaving the experts uninitiated in this practically highly relevant issue. Three meta-analyses of published literature have appeared recently, but all three have important shortcomings which limit their usefulness as a basis for nutritional recommendations [7,8,9]. The DNSG, therefore, recognized the need to update its own guidelines from 2004 [10] and to review the evidence available using the GRADE procedure to comply with current standards.

Many experts are skeptical as to the benefits of high protein diets. High animal protein intake can promote diabetes [11,12], diabetic nephropathy, and accelerate the loss of glomerular filtration rate [13,14]. Elevated levels of branched chain amino acids can increase the activity of proliferative signaling pathways, in particular the mTOR pathway [15,16], as well as increase the levels of the insulin-like growth factor 1 (IGF-1) which is an important permissive growth factor [17]. Low protein intake can even offer specific advantages by activating protective pathways associated with the integrated stress response [18]. The effect of high protein diets on life expectancy in humans is controversial. In addition to increased death from neoplastic disease, an increase in cardiovascular disease has been reported in some, but not in all, studies [19,20,21].

The following arguments are cited in favor of high protein diets: They should theoretically be advantageous as compared with carbohydrate-rich diets for people with T2D, because they cause only small increases in blood glucose due to the low carbohydrate content, they need less insulin for the metabolism of the proteins, and they increase postprandial thermogenesis [1,22,23]. This also distinguishes high protein versus high fat diets which can promote obesity due to the high energy content. The low insulin requirement of high protein diets reduces insulin-induced lipogenesis and improves blood lipids because hepatic and adipose tissues use carbohydrates for lipogenesis [24]. The lipogenesis supports the development of fatty liver and elevates circulating triglycerides, whereas the synthesis of fat from protein requires more energy, increases fat oxidation, and reduces fatty liver [22].

People with diabetes benefit from high protein diets due to better blood glucose control, and therefore less insulin is required, whether from endogenous secretion or exogenously applied. For people treated with insulin, the adequate calculation of the insulin dose becomes easier and more accurate since the possible miscalculation of the insulin dose per quantity of carbohydrate is reduced with lower quantities of carbohydrates and smaller doses of insulin. Other sources of variation, such as insulin resorption after injection or differences in carbohydrate uptake due to changes in gastric emptying or intestinal resorption due to glycemic index are also reduced [1,25,26].

Further arguments favoring high protein intake in diabetes relate to its satiation despite a relatively low energy density which is stronger and more sustained than for carbohydrates or fat [22] and which can support an adequate energy intake and help maintain a healthy body weight. In the context of weight loss diets, higher contents of protein are advocated to maintain lean body mass, and thereby improve basal metabolic rate after weight loss [22]. On the basis of these arguments, some experts and nutritionists recommend high protein diets. In addition to the diabetological arguments, high protein diets are recommended in geriatric guidelines to counteract age-related loss of muscle mass which can lead to frailty and loss of independence due to sarcopenia [27].

High protein intake, up to 35% of energy intake (E%), are advocated by some dieticians, nutritionists, doctors, and in diabetes literature, although it is difficult to achieve this intake long term [1,28,29]. The European diabetes nutrition guidelines, issued in 2004 by the DNSG as a study group of the EASD [10], recommends protein intakes between 10% to 20% of total energy intake. Recently published guidelines and recommendations by the American Diabetes Association, the Canadian Diabetes Association [6], and the British Diabetic Association [4] do not address the risks or benefits of high protein diets specifically but recommend moderate protein intakes.

The DNSG, therefore, felt that is was necessary to review the literature regarding protein intakes. High protein diets can substitute protein for carbohydrates or fat as the macronutrient. In this study, we focus on the substitution of protein for carbohydrates because most studies have used this approach and there are fewer studies where protein is substituted for fat in T2D.

## 2. Materials and Methods

The grading procedure used for quality of evidence and strength of recommendations (GRADE) [30] was based on systematic reviews and meta-analyses. Evidence grading is as follows: ⊕⊝⊝⊝ denotes very low-quality evidence, ⊕⊕⊝⊝ denotes low quality, ⊕⊕⊕⊝ denotes moderate quality, and ⊕⊕⊕⊕denotes high quality.

### 2.1. Literature Search

We performed a systematic search in Pub Med of the literature according to the following criteria:

Syntax (diabetes mellitus OR type 1 diabetes OR type 2 diabetes) AND (protein intake OR dietary protein OR high protein diet). Filter clinical trials, species: human.

We included randomized intervention studies and prospective observational studies but excluded case studies. Studies had to include participants with type 1 or T2D and had to be published in English.

### 2.2. Meta-Analysis

A meta-analysis was generated (see Supplementary Materials) using RevMan as provided by the Cochrane Foundation (http://ims.cochrane.org/revman) because the available meta-analyses did not fulfill the inclusion criteria defined by the guideline working group.

Inclusion criteria for studies to be evaluated:

- At least 50% diabetes participants
- No fewer than 20 participants or crossover design with no fewer than 10 participants
- Study duration: at least 8 weeks
- Studies achieved a documented difference in protein intake
- Studies which performed power calculations will be rated higher
- Full publication available

The PICO scheme was used for selection and evaluation according to the following criteria:

- Population: T2D, Type 1 Diabetes (T1D). Exclusion of impaired fasting glucose, impairedglucose tolerance or metabolic syndrome without manifest diabetes.
- Intervention: High protein intake in exchange for carbohydrates. High protein intake defined as >20%E protein intake per day. Studies were rated according to documented protein intakes.
- Comparator: Lower protein intake (<20% protein). Studies have to control and document differences in protein intakes achieved.
- Outcomes:
  1. Mortality, total mortality or death due to cardiovascular disease, cancer, other causes.
  2. Diabetic microvascular or macrovascular complications: diabetic nephropathy, diabetic retinopathy/maculopathy, diabetic autonomic or sensory peripheral neuropathy, cardiovascular disease (myocardial infarction and stroke), amputation, peripheral artery occlusive disease.
  3. Metabolic control: HbA1c, fasting/postprandial blood glucose, glucose excursion/variability, incidence of hypoglycemia, blood lipids (TG, HDL-C, LDL-C), inflammatory markers, blood pressure, insulin sensitivity
  4. Anthropometry: body weight, waist circumference, liver fat content.
  5. Adherence/compliance, satisfaction, Quality of Life (QoL).
  6. Decrease in medication

## 3. Results

### 3.1. Does a High Protein Diet Increase the Mortality of People with Diabetes?

High protein diets shorten lifespan in some animal models [15,16,31] and a meta-analysis of human observational studies in non-diabetic subjects reported increased all-cause mortality associated with a higher protein intake in exchange for carbohydrates [32]. Higher protein intakes were variably defined and compared upper vs. lower tertiles or quintiles of the populations but usually exceeded 20% protein of total energy intake. While there are no randomized prospective intervention studies in subjects with diabetes mellitus, observational studies provide some limited evidence: 

The European Prospective Investigation into Cancer (EPIC) study in Europe compared the mortality of 6192 people with T2D with a higher protein intake in exchange for carbohydrates over 4.4 years and did not observe an increase in mortality [33]. The ONTARGET Trial (Ongoing Telmisartan Alone and in Combination with Ramipril Global Endpoint Trial) included a substudy of 6213 diabetes participants over five years who completed food frequency questionnaires according to the modified Alternative Healthy Eating Index (mAHEI) [34]. This study had a significant mortality of 8.3% over five years but did not find any association of the higher vs. the lower tertiles of protein intake with mortality [34]. A National Health and Nutrition Examination Survey (NHANES) observational study included only a small number of diabetes participants, and therefore was excluded [15].

We concluded that no association is observed between protein intake and mortality in observational studies in people with diabetes. (quality of evidence: very low ⊕⊝⊝⊝).

### 3.2. Does a High Protein Diet Alter the Risk of Diabetic Micro- or Macrovascular Complications? 

We could not identify studies addressing this question. Diabetic nephropathy is presented in a separate section.

### 3.3. Does a High Protein Diet Improve Long Term Metabolic Control in People with T2D Mellitus Underweight-Stable Conditions?

People with T2D mellitus and younger than 65 years can consume 10% to 20% of E% as protein, and 15% to 20% of E% as protein for people older than 65 years of age, corresponding to approximately 0.8 to 1.2 g/kg body weight in women and 0.9 to 1.3 g/kg body weight in men under isocaloric conditions. ⊕⊕⊝⊝, low-quality evidence. 

#### Commentary 

Minimal thresholds of protein intake have not been investigated in people with diabetes. The usual and general recommendation of a minimum intake of 0.8 g/kg bodyweight per day or about 10% of E% is based on studies of nitrogen balance in healthy subjects [35,36]. The analysis of currently available studies shows that there are very limited data on protein intakes of less than 15% of E%, corresponding to about 1 g/kg body weight per day and a short study with 10% of E% as protein indicated that there can be an increased risk of sarcopenia in older subjects with T2D [37]. There is only one study which investigated a higher protein intake over two years in 419 people with T2D [38]. Although this study targeted a protein intake of 30% of E% in the high protein group, the achieved protein intake was 21% of E% in the high protein group. The low protein group, which was intended to consume 15% of E% as protein, in fact consumed about 20% of E% as protein. This study showed no differences in metabolic parameters between the groups, as might be expected. The study did not report adverse events over two years. The two above-mentioned observational studies, which included about 6200 subjects, each achieved an estimated amount of 20% of E% as protein intake in the higher protein tertiles or groups but did not specifically analyze subgroups with a higher protein intake [33,34]. Since no data are published on long-term (>two years) intake of higher protein, and since some human data from nondiabetic subjects [15,20], as well as data from animal studies [16,31], indicate potential risk, we recommend a maximum intake of 20% of E% as protein.

There is also a lack of randomized studies with regard to lower protein intake. Geriatric experts of the PROT-AGE group recommend higher protein intakes to reduce the risk of frailty and sarcopenia in the older population which often applies to people with T2D [27,37]. Therefore, a range of 15% to 20% of E% as protein intake appears to be reasonable during the long term. The risk of sarcopenia in the older population has not been sufficiently clarified in people with diabetes and is likely to be underestimated, especially after periods of inactivity or treatment with glucocorticoids which can enhance rapid loss of muscle mass and strength [27].

### 3.4. Does a High, as Compared with a Standard, Protein Diet Lead to Better Metabolic Control in People with T2D Mellitus and a GFR >60 mL/min/1.73 m^2^ in Hypocaloric Weight Loss Diets?

People with T2D mellitus can consume 23% to 32% of E% as protein short term (up to 12 months) to achieve weight loss. Weight reduction is related to significant improvements in HbA1c, independent of protein intake. (⊕⊕⊝⊝, low-quality evidence).

People with T2D mellitus can consume 23% to 32% of E% as protein in the short term to improve fasting blood glucose. (⊕⊝⊝⊝ very low-quality evidence).

Blood lipids are not improved by higher (23% to 32% of E% as protein short term) as compared with lower protein weight loss diets. (⊕⊕⊝⊝ low-quality evidence).

Higher protein diets from 23% to 32% of E% may be administered in people with T2D for up to one year to improve systolic and diastolic blood pressure. (⊕⊕⊝⊝, low-quality evidence).

#### 3.4.1. Commentary

High protein weight loss diets are popular and claim to reduce hunger and to prevent the loss of muscle mass. People with T2D are mostly overweight and desire weight loss, which is also recommended for obesity as a treatment strategy. Indeed, virtually all studies of high protein diets were combined with energy restriction. The studies are of shorter duration at between six to 64 weeks and achieved high protein intakes of 27% to 32% in the high protein groups as compared with 17% to 20% of E% in the lower protein groups. If total caloric intake is reduced, the absolute amount of protein in a high protein diet becomes smaller and ranges between 87 and140 g/day with an estimated protein intake of 0.9 to 1.2 g/kg/day. High protein intake in the context of hypocaloric diets, therefore, corresponds quantitatively to normal absolute protein intake. 

To address the role of protein intake in T1D or T2D, we performed a meta-analysis based on the GRADE procedure using the Cochrane Foundation’s Review Manager Version 5.1 (http://hiv.cochrane.org/sites/hiv.cochrane.org/files/uploads/rm5tutorial.pdf) which is presented in the Supplementary Information. Studies were selected according to the PICO criteria defined above. Although three meta-analyses have been published addressing the effects of protein intake in T2DM, they did not correspond to the PICO criteria defined by the DNSG study group. One meta-analysis [7] included nine studies in 418 type 2 diabetic subjects with a duration of 24 to 54 weeks. The intervention groups targeted 25% to 32% of E% as protein and the control groups had 15% to 20% of E% as protein in the study design. Five of the nine studies substituted protein for carbohydrate while four studies [29,39,40,41] included additional changes in fat intake. A second meta-analysis [9] included only two weight-loss, high protein studies which lasted 52 [42] and 64 weeks [43]. The third meta-analysis did not take into consideration whether studies achieved differences in protein intake in the intervention groups [8]. 

#### 3.4.2. HbA1c

While weight loss reproducibly reduced HbA1c in all studies, there was no significant difference between the high and low protein groups (0.1% or 1.09 mmol/L greater reduction of HbA1c in the high protein group, see Supplementary Materials). The published meta-analyses reported a significant reduction of −0.52% or −5.7 mmol/L [7] and −0.28% or −3.1 mmol/l HbA1c [9]. Both studies reported significant heterogeneity (I^2^ = 57.2% and I^2^ = 60%) which indicates high variation in the outcome. Moreover, since greater weight loss occurred in the high protein groups, the greater reductions in HbA1c could be attributable to greater weight loss. The third meta-analysis did not find significant differences [8]. 

We concluded that significant improvements in HbA1c are achieved with weight-loss diets which are not affected by higher protein diets. 

#### 3.4.3. Fasting Blood Glucose 

Fasting blood glucose was reduced by weight loss in the control and high protein groups with a nonsignificant greater reduction of 13.0 mg/dL or 0.72 mmol/L in the high protein group. Similar results were reported in another meta-analysis [7]. 

#### 3.4.4. Blood Lipids 

The meta-analysis did not show significant differences between the high and low protein groups regarding HDL-, or LDL- cholesterol and there was no heterogeneity (I^2^ = 0%) in the meta-analyses. Similar results were reported in the other meta-analyses [7,8]. 

Triglycerides (TG) were investigated in the meta-analysis by Dong et al. [7] and in the study by Luger et al. [44] and neither found significant differences between the groups. However, it should be noted that there is no clear association between fasting TG and CVD, whereas there is an association between high non-fasting TG and CVD [45]. 

We concluded that blood lipids are not improved by higher as compared with lower protein weight-loss diets. 

#### 3.4.5. Blood Pressure 

A significantly greater reduction in systolic blood pressure by −4.41 mm Hg was observed in the high protein group which had a low heterogeneity (I^2^ = 0%). Diastolic blood pressure similarly showed a significantly greater decline of −3.61 (*p* = 0.008) mm Hg in the high protein group, with a low heterogeneity between the four studies (I^2^ = 27%, *p* = 0.25). Similar results were calculated in the meta-analysis of Dong et al. [7], whereas Ajala et al. [9] did not analyze this parameter.

We concluded that higher protein weight-loss diets caused a significantly greater reduction of systolic and diastolic blood pressure than lower protein diets in people with T2D. There is no information on metabolic outcomes in weight-stable subjects with T2D.

### 3.5. Does a High Protein Diet, as Compared with a Low Protein Diet, Promote Greater Weight Loss in People with T2D? 

Weight loss was achieved in the control and high protein groups in all studies. However, greater weight loss of −1.12 kg (*p* = 0.01) occurred in the high protein group in the meta-analysis with a low heterogeneity between the studies (I^2^ = 5%, see Supplementary Materials). The published meta-analyses either observed a significantly greater weight loss in the high protein group [7] or no difference [8,9].

We concluded that a moderately greater weight loss is achieved with higher as compared with lower protein hypocaloric diets in T2DM.

Values and preferences: The data show a modest improvement of metabolism and a slightly greater weight reduction with the use of high protein weight loss diets as compared with standard protein weight loss diets on average. Meta-analyses do not capture individual preferences and responses, and the success of a specific dietary approach depends on the acceptance by the individual and their ability to adhere to the dietary approach. Therefore, the diets can be tried and tested as a potentially useful approach to initiate, motivate, and support the individual to achieve weight loss and metabolic improvement. There is no indication of harm due to the higher protein intake. The dietary options should, therefore, be adapted to the individual values and preferences.

### 3.6. Does Plant Protein, as Compared with Animal Protein or Red versus White Meat, have Different Effects on Metabolic Control in People with Diabetes Mellitus?

There is no sufficient evidence suggesting advantages of specific types of protein.

#### Commentary

There are few studies on the effect of different types of protein on metabolic control in diabetes and the studies are short term, with small numbers of participants.

The longest study included 41 participants with type 2 diabetes and nephropathy. The plant protein diet contained 65% plant protein, including 30% soy protein and 35% animal protein, while the control diet contained 30% plant and 70% animal protein. The animal protein was not defined in detail and the content of milk protein was unclear. The HbA1c values were not reported, but fasting glucose was improved with the plant protein diet. There were no effects on glomerular filtration rates, TG, and HDL cholesterol while total and LDL-cholesterol were lowered by the plant protein diet [46].

Four studies showed effects on lipid metabolism. A diet rich in plant protein reduced either total cholesterol or LDL cholesterol or apolipoprotein B in subjects with microalbuminuria. A positive effect on glucose control was not observed.

A randomized study in 15 subjects with T1D used a crossover design with three weeks of a low-protein diet of 0.5 g/kg body weight, primarily plant and milk protein, followed by a test diet with normal protein content based on chicken and fish protein, and a third phase based on normal protein content composed of 80% red meat and 20% chicken. The diet with red meat led to higher total cholesterol levels as compared with the low-protein or chicken and fish protein diets. The glomerular filtration rate was reduced with the low-protein or chicken and fish protein diets which was interpreted as a reduced hyperfiltration, and thus a positive effect [47].

A further study in 23 T2D subjects compared six weeks of a plant protein diet with six weeks of a diet containing 60% animal and 40% plant protein in a crossover design. This study also reported reduced total cholesterol in the presence of plant protein and a lower systolic blood pressure as compared with the animal protein intervention phase [48].

We concluded that diets rich in plant protein, white meat, or fish protein, as compared with red meat protein, improve the levels of total cholesterol in people with T2D.

### 3.7. Does A Reduction in Protein Intake Reduce the Loss of Glomerular Filtration Rate in People with Diabetes Mellitus and Moderate Renal Impairment,/GFR <60 mL/min/and >45 mL/min

Maintaining protein intake in the normal range of about 0.8 g/kg body weight in people with diabetic nephropathy and a glomerular filtration rate below 60 ml/min and above 45 ml/min did not lead to faster decline of renal function. (2 | ⊕⊕⊝⊝)

#### Commentary

The question whether reduced protein intake slows the progression of diabetic nephropathy and reduces the rate of loss of glomerular filtration rate has a long history, in particular because animal models show strong effects of protein intake [14]. Numerous clinical studies and meta-analyses and reviews have been published. However, the results are still controversial. A systematic review from the Cochrane Collaboration by Robertson and co-workers [49] summarized 12 studies comprising nine RCTs and three follow-up studies including a total of 585 people with diabetes mellitus (322 T1D and 263 T2D). A comparison with a normal protein diet (1 to 2 g/kg bodyweight per day) showed that a low protein diet (0.3 to 0.8 g/kg per day) slightly reduced the decline in glomerular filtration rate but the effect was not significant.

A systematic review by Nezu and co-workers [13] included 13 randomized studies comprising 779 subjects with T1D and T2D and only evaluated studies which achieved a documented difference in protein intake during the studies. The low protein diets significantly reduced the decline in glomerular filtration rates in studies in which the compliance was high. However, an evaluation of the study according to GRADE criteria led to a low evidence rating, such that no strong recommendation can be deduced from these studies.

Our meta-analysis did not show a significant effect of low protein as compared with normal protein diets on the decline of GFR in 478 subjects included in nine studies and no effect on proteinuria in 11 studies including 488 participants (see Supplementary Materials).

A primary problem of most studies is the poor compliance of study subjects with the recommendation of lower protein intake. Only very few studies achieved protein intakes of 0.6 g/kg body weight per day and the reported intake was usually around 0.8 g/kg bodyweight per day or even higher. The studies address this problem and report that compliance was poor, reflecting the practical problems in maintaining a low protein diet. There were also methodological problems in controlling food intake in the different randomized studies which made comparisons quite difficult. 

There are two randomized controlled studies which evaluated the effects of high protein intake in people with T2D on kidney function. One study investigated 12 T2D subjects with microalbuminuria in a crossover design over three weeks each. The high protein intervention was achieved by administering supplements and protein intake was 2 g/kg bodyweight per day. The control group was targeted at 0.8 g/kg bodyweight per day, but effectively achieved 1.1 g/kg body weight per day protein intake. Compliance was well controlled and complete dietary plans were administered. The high protein diet did not lead to a decrease in glomerular filtration rate while the control diet led to a reduction from 2.18 +/− 0.77 to 1.5 +/− 0.8 mL/sec/1.73 m^2^ (*p* < 0.05).

In a recent study by Pedersen and Jesudason et al. [50,51], a hypocaloric high-protein diet was compared with a hypocaloric low-protein diet. The inclusion criteria were a microalbuminuria or impaired renal function. There were no significant differences in glomerular filtration rate between the intervention and control groups. Both groups had a significant weight loss which improved renal function. The fasting blood glucose, total cholesterol, TG, and HDL cholesterol were significantly reduced by the weight loss, but there were no differences according to diet. The HbA1c showed a significantly greater reduction in the high protein diet. The albuminuria was not negatively affected by the high protein diet. Only a few study participants had a glomerular filtration rate below 60 ml/min. These participants did not experience a reduction in glomerular filtration rate, while subjects with a hyperfiltration showed a normalization of the glomerular filtration rate. Similar results were reported by Tirosh and coworkers in 45 study participants with T2D in the Direct study over two years. The diet containing 22% of E% as protein diet did not impair the GFR or worsen the albuminuria, while most of the improvement in GFR was associated with the weight loss [52].

A large observational study, the ONTARGET Study [34], evaluated the effect of nutrition on renal function in 6200 participants with T2D and high cardiovascular risk over five years by administering food frequency questionnaires. Twenty per cent of the participants had a decline in glomerular filtration rate of 5% per year. Participants with the highest protein intake showed a significantly slower decline in GFR than the tertile with low protein intake. This overall high-risk group of study participants with diabetes had a mortality of 8.3% over five years and the high protein intake had no effect on mortality.

Overall, negative effects of a high protein intake on the glomerular filtration rate could not be established, and therefore a moderate protein intake of 0.8 g/kg body weight in people with impaired renal function, appears adequate and is compatible with a normal lifestyle.

### 3.8. Is There Evidence That High Protein Diets Increase Diabetic Retinopathy: Maculopathy or Neuropathy 

There are no controlled studies of sufficient length and quality to answer this question [53].

## Supplementary Materials

The following are available online at https://www.mdpi.com/2072-6643/12/2/365/s1.

## Abbreviations

EPIC—European prospective investigation into cancer
HDL-C—high density lipoprotein cholesterol
IGF1—insulin-like growth factor 1
LDL-C—low density lipoprotein cholesterol
NHANES—National Health and Nutrition Examination Survey
mAHEI—modified alternative healthy eating index
mTOR—mammalian target of rapamycin TG
QoL—quality of life
T1D—type 1 diabetes mellitus
T2D—type 2 diabetes mellitus
TG—triglyceride

## References

[1] Feinman R.D., Pogozelski W.K., Astrup A., Bernstein R.K., Fine E.J., Westman E.C., Accurso A., Frassetto L., Gower B.A., McFarlane S.I. (2015). Dietary carbohydrate restriction as the first approach in diabetes management: Critical review and evidence base. Nutrition.

[2] Nuttall F.Q., Gannon M.C. (2006). The metabolic response to a high-protein, low-carbohydrate diet in men with type 2 diabetes mellitus. Metabolism.

[3] Leidy H.J., Clifton P.M., Astrup A., Wycherley T.P., Westerterp-Plantenga M.S., Luscombe-Marsh N.D., Woods S.C., Mattes R.D. (2015). The role of protein in weight loss and maintenance. Am. J. Clin. Nutr..

[4] Diabetes UK (2011). Evidence-Based Nutrition Guidelines for the Prevention and Management of Diabetes. http://www.diabetes.org.uk/Documents/Reports/Nutritional_guidelines200911.pdf.

[5] Evert A.B., Boucher J.L., Cypress M., Dunbar S.A., Franz M.J., Mayer-Davis E.J., Neumiller J.J., Nwankwo R., Verdi C.L., Urbanski P. (2014). Nutrition therapy recommendations for the management of adults with diabetes. Diabetes Care.

[6] Committee C.D.A.C.P.G.E., Dworatzek P.D., Arcudi K., Gougeon R., Husein N., Sievenpiper J.L., Williams S.L. (2013). Nutrition therapy. Can J. Diabetes.

[7] Dong J.Y., Zhang Z.L., Wang P.Y., Qin L.Q. (2013). Effects of high-protein diets on body weight, glycaemic control, blood lipids and blood pressure in type 2 diabetes: Meta-analysis of randomised controlled trials. Br. J. Nutr..

[8] Naude C.E., Schoonees A., Senekal M., Young T., Garner P., Volmink J. (2014). Low carbohydrate versus isoenergetic balanced diets for reducing weight and cardiovascular risk: A systematic review and meta-analysis. PLoS ONE.

[9] Ajala O., English P., Pinkney J. (2013). Systematic review and meta-analysis of different dietary approaches to the management of type 2 diabetes. Am. J. Clin. Nutr..

[10] Mann J.I., De Leeuw I., Hermansen K., Karamanos B., Karlstrom B., Katsilambros N., Riccardi G., Rivellese A.A., Rizkalla S., Slama G. (2004). Evidence-based nutritional approaches to the treatment and prevention of diabetes mellitus. Nutr. Metab. Cardiovasc. Dis..

[11] Shang X., Scott D., Hodge A.M., English D.R., Giles G.G., Ebeling P.R., Sanders K.M. (2016). Dietary protein intake and risk of type 2 diabetes: Results from the Melbourne Collaborative Cohort Study and a meta-analysis of prospective studies. Am. J. Clin. Nutr..

[12] Van Nielen M., Feskens E.J., Mensink M., Sluijs I., Molina E., Amiano P., Ardanaz E., Balkau B., Beulens J.W., Boeing H. (2014). Dietary protein intake and incidence of type 2 diabetes in Europe: The EPIC-InterAct Case-Cohort Study. Diabetes Care.

[13] Nezu U., Kamiyama H., Kondo Y., Sakuma M., Morimoto T., Ueda S. (2013). Effect of low-protein diet on kidney function in diabetic nephropathy: Meta-analysis of randomised controlled trials. BMJ Open.

[14] Fouque D., Aparicio M. (2007). Eleven reasons to control the protein intake of patients with chronic kidney disease. Nat. Clin. Pract. Nephrol..

[15] Levine M.E., Suarez J.A., Brandhorst S., Balasubramanian P., Cheng C.W., Madia F., Fontana L., Mirisola M.G., Guevara-Aguirre J., Wan J. (2014). Low Protein Intake Is Associated with a Major Reduction in IGF-1, Cancer, and Overall Mortality in the 65 and Younger but Not Older Population. Cell Metab..

[16] Solon-Biet S.M., McMahon A.C., Ballard J.W., Ruohonen K., Wu L.E., Cogger V.C., Warren A., Huang X., Pichaud N., Melvin R.G. (2014). The ratio of macronutrients, not caloric intake, dictates cardiometabolic health, aging, and longevity in ad libitum-fed mice. Cell Metab..

[17] Lopez-Otin C., Blasco M.A., Partridge L., Serrano M., Kroemer G. (2013). The hallmarks of aging. Cell.

[18] Galluzzi L., Pietrocola F., Levine B., Kroemer G. (2014). Metabolic Control of Autophagy. Cell.

[19] Halton T.L., Willett W.C., Liu S., Manson J.E., Albert C.M., Rexrode K., Hu F.B. (2006). Low-carbohydrate-diet score and the risk of coronary heart disease in women. N. Engl. J. Med..

[20] Lagiou P., Sandin S., Lof M., Trichopoulos D., Adami H.O., Weiderpass E. (2012). Low carbohydrate-high protein diet and incidence of cardiovascular diseases in Swedish women: Prospective cohort study. BMJ.

[21] Song M., Fung T.T., Hu F.B., Willett W.C., Longo V.D., Chan A.T., Giovannucci E.L. (2016). Association of Animal and Plant Protein Intake with All-Cause and Cause-Specific Mortality. JAMA Intern Med..

[22] Westerterp-Plantenga M.S., Lemmens S.G., Westerterp K.R. (2012). Dietary protein—its role in satiety, energetics, weight loss and health. Br. J. Nutr..

[23] Nuttall F.Q., Gannon M.C. (2004). Metabolic response of people with type 2 diabetes to a high protein diet. Nutr. Metab..

[24] Ameer F., Scandiuzzi L., Hasnain S., Kalbacher H., Zaidi N. (2014). De novo lipogenesis in health and disease. Metabolism.

[25] Waldron S. (1996). Current controversies in the dietary management of diabetes in childhood and adolescence. Br. J. Hosp. Med..

[26] Nielsen J.V., Gando C., Joensson E., Paulsson C. (2012). Low carbohydrate diet in type 1 diabetes, long-term improvement and adherence: A clinical audit. Diabetol. Metab. Syndr..

[27] Bauer J., Biolo G., Cederholm T., Cesari M., Cruz-Jentoft A.J., Morley J.E., Phillips S., Sieber C., Stehle P., Teta D. (2013). Evidence-based recommendations for optimal dietary protein intake in older people: A position paper from the PROT-AGE Study Group. J. Am. Med. Dir. Assoc..

[28] Ballard K.D., Quann E.E., Kupchak B.R., Volk B.M., Kawiecki D.M., Fernandez M.L., Seip R.L., Maresh C.M., Kraemer W.J., Volek J.S. (2013). Dietary carbohydrate restriction improves insulin sensitivity, blood pressure, microvascular function, and cellular adhesion markers in individuals taking statins. Nutr. Res..

[29] Gannon M.C., Nuttall F.Q. (2004). Effect of a high-protein, low-carbohydrate diet on blood glucose control in people with type 2 diabetes. Diabetes.

[30] Brozek J.L., Akl E.A., Alonso-Coello P., Lang D., Jaeschke R., Williams J.W., Phillips B., Lelgemann M., Lethaby A., Bousquet J. (2009). Grading quality of evidence and strength of recommendations in clinical practice guidelines. Part 1 of 3. An overview of the GRADE approach and grading quality of evidence about interventions. Allergy.

[31] Partridge L., Alic N., Bjedov I., Piper M.D. (2011). Ageing in Drosophila: The role of the insulin/Igf and TOR signalling network. Exp. Gerontol..

[32] Noto H., Goto A., Tsujimoto T., Noda M. (2013). Low-carbohydrate diets and all-cause mortality: A systematic review and meta-analysis of observational studies. PLoS ONE.

[33] Burger K.N., Beulens J.W., van der Schouw Y.T., Sluijs I., Spijkerman A.M., Sluik D., Boeing H., Kaaks R., Teucher B., Dethlefsen C. (2012). Dietary fiber, carbohydrate quality and quantity, and mortality risk of individuals with diabetes mellitus. PLoS ONE.

[34] Dunkler D., Dehghan M., Teo K.K., Heinze G., Gao P., Kohl M., Clase C.M., Mann J.F., Yusuf S., Oberbauer R. (2013). Diet and kidney disease in high-risk individuals with type 2 diabetes mellitus. JAMA Intern. Med..

[35] Wu G. (2016). Dietary protein intake and human health. Food Funct..

[36] Golden M., Waterlow J.C., Picou D. (1977). The relationship between dietary intake, weight change, nitrogen balance, and protein turnover in man. Am. J. Clin. Nutr..

[37] Labonte C.C., Chevalier S., Marliss E.B., Morais J.A., Gougeon R. (2015). Effect of 10% dietary protein intake on whole body protein kinetics in type 2 diabetic adults. Clin. Nutr..

[38] Krebs J.D., Elley C.R., Parry-Strong A., Lunt H., Drury P.L., Bell D.A., Robinson E., Moyes S.A., Mann J.I. (2012). The Diabetes Excess Weight Loss (DEWL) Trial: A randomised controlled trial of high-protein versus high-carbohydrate diets over 2 years in type 2 diabetes. Diabetologia.

[39] Daly M.E., Paisey R., Paisey R., Millward B.A., Eccles C., Williams K., Hammersley S., MacLeod K.M., Gale T.J. (2006). Short-term effects of severe dietary carbohydrate-restriction advice in Type 2 diabetes—A randomized controlled trial. Diabet. Med..

[40] Papakonstantinou E., Triantafillidou D., Panagiotakos D.B., Koutsovasilis A., Saliaris M., Manolis A., Melidonis A., Zampelas A. (2010). A high-protein low-fat diet is more effective in improving blood pressure and triglycerides in calorie-restricted obese individuals with newly diagnosed type 2 diabetes. Eur. J. Clin. Nutr..

[41] Westman E.C., Yancy W.S., Mavropoulos J.C., Marquart M., McDuffie J.R. (2008). The effect of a low-carbohydrate, ketogenic diet versus a low-glycemic index diet on glycemic control in type 2 diabetes mellitus. Nutr. Metab. (Lond).

[42] Larsen R.N., Mann N.J., Maclean E., Shaw J.E. (2011). The effect of high-protein, low-carbohydrate diets in the treatment of type 2 diabetes: A 12 month randomised controlled trial. Diabetologia.

[43] Brinkworth G.D., Noakes M., Parker B., Foster P., Clifton P.M. (2004). Long-term effects of advice to consume a high-protein, low-fat diet, rather than a conventional weight-loss diet, in obese adults with type 2 diabetes: One-year follow-up of a randomised trial. Diabetologia.

[44] Luger M., Holstein B., Schindler K., Kruschitz R., Ludvik B. (2013). Feasibility and efficacy of an isocaloric high-protein vs. standard diet on insulin requirement, body weight and metabolic parameters in patients with type 2 diabetes on insulin therapy. Exp. Clin. Endocrinol. Diabetes.

[45] Bansal S., Buring J.E., Rifai N., Mora S., Sacks F.M., Ridker P.M. (2007). Fasting compared with nonfasting triglycerides and risk of cardiovascular events in women. JAMA.

[46] Azadbakht L., Atabak S., Esmaillzadeh A. (2008). Soy protein intake, cardiorenal indices, and C-reactive protein in type 2 diabetes with nephropathy: A longitudinal randomized clinical trial. Diabetes Care.

[47] Pecis M., de Azevedo M.J., Gross J.L. (1994). Chicken and fish diet reduces glomerular hyperfiltration in IDDM patients. Diabetes Care.

[48] Wheeler M.L., Fineberg S.E., Fineberg N.S., Gibson R.G., Hackward L.L. (2002). Animal versus plant protein meals in individuals with type 2 diabetes and microalbuminuria: Effects on renal, glycemic, and lipid parameters. Diabetes Care.

[49] Robertson L., Waugh N., Robertson A. (2007). Protein restriction for diabetic renal disease. Cochrane Database Syst. Rev..

[50] Pedersen E., Jesudason D.R., Clifton P.M. (2013). High protein weight loss diets in obese subjects with type 2 diabetes mellitus. Nutr. Metab. Cardiovasc. Dis..

[51] Jesudason D.R., Pedersen E., Clifton P.M. (2013). Weight-loss diets in people with type 2 diabetes and renal disease: A randomized controlled trial of the effect of different dietary protein amounts. Am. J. Clin. Nutr..

[52] Tirosh A., Golan R., Harman-Boehm I., Henkin Y., Schwarzfuchs D., Rudich A., Kovsan J., Fiedler G.M., Bluher M., Stumvoll M. (2013). Renal function following three distinct weight loss dietary strategies during 2 years of a randomized controlled trial. Diabetes Care.

[53] Wycherley T.P., Moran L.J., Clifton P.M., Noakes M., Brinkworth G.D. (2012). Effects of energy-restricted high-protein, low-fat compared with standard-protein, low-fat diets: A meta-analysis of randomized controlled trials. Am. J. Clin. Nutr..

