# Supplementary material for: The Effects of Different Quantities and Qualities of Protein Intake in People with Diabetes Mellitus
Source: Nutrients. 2020 Jan 30;12(2):365. doi: 10.3390/nu12020365 (PMC7071151; doi:10.3390/nu12020365)
Supplement: Supplementary file 1 [file nutrients-12-00365-s001.pdf]

Supplementary Materials

Table of contents

1 Meta-analysis - High protein diets in diabetes patients 2
1.1 Weight loss (kg) 2
1.2 Fasting blood glucose (mmol/l) 3
1.3 HbA1c (%) 4
1.4 High density lipoprotein (mmol/l) 5
1.5 Low density lipoprotein (mmol/l) 6
1.6 Systolic blood pressure (mmHg) 7
1.7 Diastolic blood pressure (mmHg) 8
2 Meta-analysis - Low protein diets in diabetic nephropathy 9
2.1 Glomerular filtration rate (ml/min/1,73m²) 9
2.2 Proteinuria 10
3 Tables of evidence 11
3.1 High protein diet in patients with type 2 diabetes mellitus (RCTs) 11
3.2 Overview of nutrient intake in high protein diet 11
3.3 Working document – Results of high protein diets in diabetes patients 13
3.4 Excluded RCTs 14
3.5 Different types of protein in patients with type 2 diabetes mellitus (RCT) 16
3.6 Low protein intake in diabetic nephropathy (RCT) 19
3.7 Low protein intake in diabetic nephropathy (existing meta-analysis) 21
3.8 Working document – Results of low protein diet in diabetic nephropathy 23
4 GRADE 24
4.1 Summary of findings table - high protein diets in diabetes patients 24
4.2 GRADE profile – high protein diets in diabetes patients 26
4.3 Summary of findings table – low protein diets in diabetic nephropathy 28
4.4 GRADE profile – low protein diets in diabetic nephropathy 30
5 Literature 31

1 Meta-analysis - High protein diets in diabetes patients

1.1 Weight loss (kg)

Figure 1: Results of the meta-analysis - Effect of high protein diet on weight loss in diabetes patients

Study or Subgroup	Mean	SD	Total	Mean	SD	Total	Weight	IV, Random, 95% CI	A	B	C	D	E	F	G	H	I	J	K	L
Brinkworth et al. (2004)	-3.7	4.36	19	-2.2	4.79	19	10.6%	-1.50 [-4.41, 1.41]	+	+	-	-	-	+	+	-	-			
Larsen et al. (2011)	-2.2321	3.779	53	-2.1652	4.2655	46	32.6%	-0.07 [-1.67, 1.53]	+	+	-	-		+	+	+	+			
Luger et al. (2013)	-3.09	2.44	21	-1.01	2.05	22	43.9%	-2.08 [-3.43, -0.73]	+	+	-	-		+	+	+	+			
Pedersen et al. (2013)	-9.7	13.36	21	-6.6	7.1	24	2.3%	-3.10 [-9.48, 3.28]	+	+	-	-	+	+	+	-	-			
Wycherley et al. (2010)	-9	4.8	21	-8.6	4.6	19	10.6%	-0.40 [-3.31, 2.51]	+	+	-	-		+	+	-	+			
**Total (95% CI)**			**135**			**130**	**100.0%**	**-1.21 [-2.17, -0.24]**												

Heterogeneity: Tau² = 0.08; Chi² = 4.23, df = 4 (P = 0.38); I² = 5%
Test for overall effect: Z = 2.46 (P = 0.01)

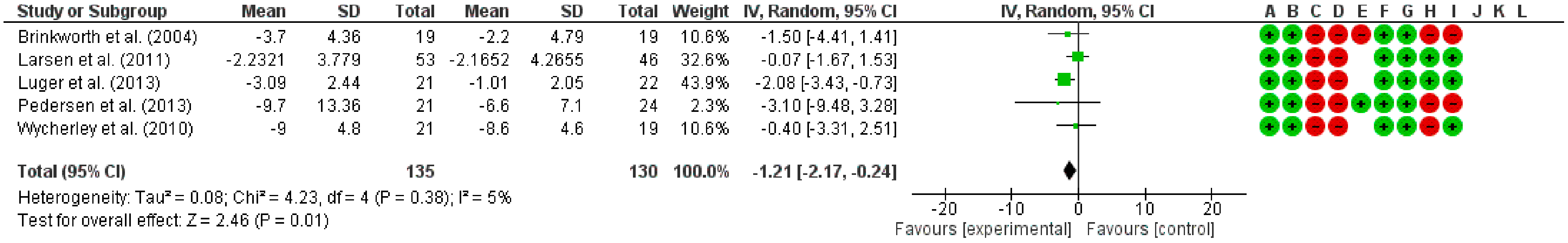

Risk of bias legend
(A) Clearly focused research question
(B) Random sequence generation (selection bias)
(C) Allocation concealment (selection bias)
(D) Blinding of participants and personnel (performance bias)
(E) Blinding of outcome assessment (detection bias)
(F) Groups are similar at the start
(G) Standardised, valid and reliable values
(H) Drop out rate
(I) Intention-to-treat analysis
(J) Incomplete outcome data (attrition bias)
(K) Selective reporting (reporting bias)
(L) Other bias

1.2 Fasting blood glucose (mmol/l)

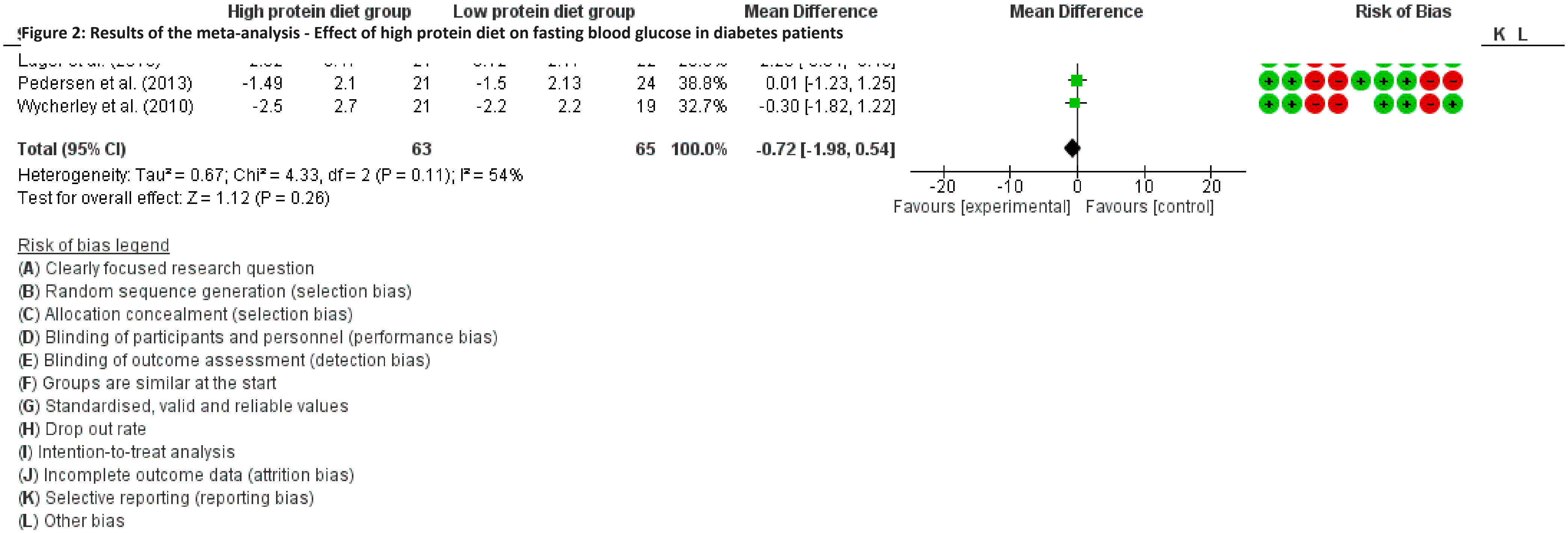

Figure 2: Results of the meta-analysis - Effect of high protein diet on fasting blood glucose in diabetes patients

1.3 HbA1c (%)

	High protein diet group			Low protein diet group				Mean Difference	Risk of Bias											
Study or Subgroup	Mean	SD	Total	Mean	SD	Total	Weight	IV, Random, 95% CI	A	B	C	D	E	F	G	H	I	J	K	L
Larsen et al. (2011)	-0.234	1.0637	53	-0.2783	0.9944	46	31.8%	0.04 [-0.36, 0.45]	+	+	−	−		+	+	+	+			
Luger et al. (2013)	-0.25	0.56	21	-0.19	0.6	22	42.9%	-0.06 [-0.41, 0.29]	+	+	−	−		+	+	+	+			
Pedersen et al. (2013)	-0.352	1.12	21	-0.25	0.87	24	15.3%	-0.10 [-0.69, 0.49]	+	+	−	−	+	+	+	−	−			
Wycherley et al. (2010)	-1.8	1.6	21	-1.1	0.6	19	10.0%	-0.70 [-1.44, 0.04]	+	+	−	−		+	+	−	+			
**Total (95% CI)**			**116**			**111**	**100.0%**	**-0.10 [-0.33, 0.14]**												

Heterogeneity: Tau² = 0.00; Chi² = 3.09, df = 3 (P = 0.38); I² = 3%
Test for overall effect: Z = 0.81 (P = 0.42)

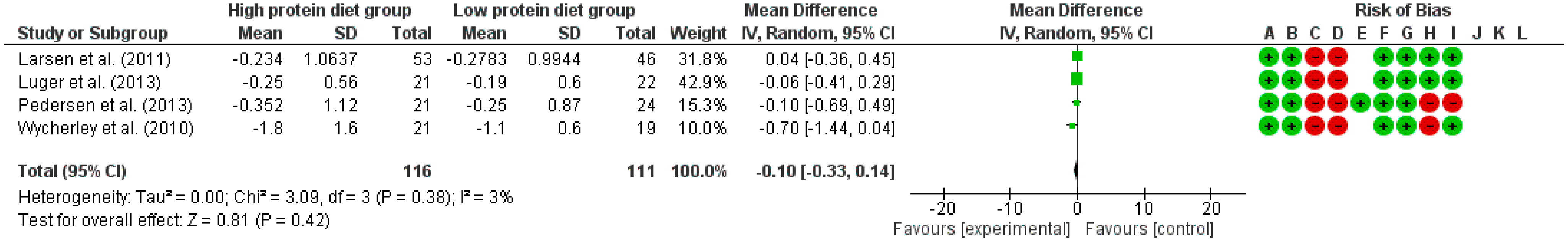

Risk of bias legend
(A) Clearly focused research question
(B) Random sequence generation (selection bias)
(C) Allocation concealment (selection bias)
(D) Blinding of participants and personnel (performance bias)
(E) Blinding of outcome assessment (detection bias)
(F) Groups are similar at the start
(G) Standardised, valid and reliable values
(H) Drop out rate
(I) Intention-to-treat analysis
(J) Incomplete outcome data (attrition bias)
(K) Selective reporting (reporting bias)
(L) Other bias

Figure 3: Results of the meta-analysis - Effect of high protein diet on hbA1c in diabetes patients

1.4 High density lipoprotein (mmol/l)

Study or Subgroup	High protein diet group Mean	SD	Total	Low protein diet group Mean	SD	Total	Weight	Mean Difference IV, Random, 95% CI	A	B	C	D	E	F	G	H	I	J	K	L
Larsen et al. (2011)	0.0813	0.2916	53	0.0757	0.2298	46	27.2%	0.01 [-0.10, 0.11]	+	+	−	−		+	+	+	+			
Luger et al. (2013)	0.02	0.18	21	0.04	0.15	21	28.7%	-0.02 [-0.12, 0.08]	+	+	−	−		+	+	+	+			
Pedersen et al. (2013)	0.114	0.127	21	0.083	0.229	24	25.4%	0.03 [-0.08, 0.14]	+	+	−	−	+	+	+	−	−			
Wycherley et al. (2010)	-0.1	0.2	21	0	0.2	19	18.7%	-0.10 [-0.22, 0.02]	+	+	−	−		+	+	−	+			
**Total (95% CI)**			**116**			**110**	**100.0%**	**-0.02 [-0.07, 0.04]**												

Heterogeneity: Tau² = 0.00; Chi² = 2.68, df = 3 (P = 0.44); I² = 0%
Test for overall effect: Z = 0.55 (P = 0.58)

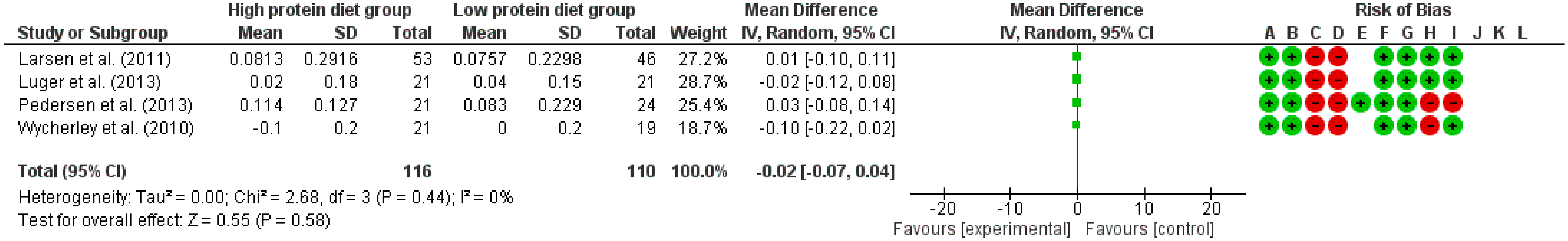

Risk of bias legend
(**A**) Clearly focused research question
(**B**) Random sequence generation (selection bias)
(**C**) Allocation concealment (selection bias)
(**D**) Blinding of participants and personnel (performance bias)
(**E**) Blinding of outcome assessment (detection bias)
(**F**) Groups are similar at the start
(**G**) Standardised, valid and reliable values
(**H**) Drop out rate
(**I**) Intention-to-treat analysis
(**J**) Incomplete outcome data (attrition bias)
(**K**) Selective reporting (reporting bias)
(**L**) Other bias

Figure 4: Results of the meta-analysis - Effect of high protein diet on high density lipoprotein in diabetes patients

1.5 Low density lipoprotein (mmol/l)

	High protein diet group			Low protein diet group				Mean Difference	Risk of Bias											
Study or Subgroup	Mean	SD	Total	Mean	SD	Total	Weight	IV, Random, 95% CI	A	B	C	D	E	F	G	H	I	J	K	L
Larsen et al. (2011)	-0.0521	0.58878	53	0.0444	0.7622	46	41.2%	-0.10 [-0.37, 0.17]	+	+	−	−		+	+	+	+			
Luger et al. (2013)	-0.11	0.71	21	-0.05	0.71	21	16.5%	-0.06 [-0.49, 0.37]	+	+	−	−		+	+	+	+			
Pedersen et al. (2013)	0.196	0.8662	21	0.009	0.5587	24	16.2%	0.19 [-0.25, 0.62]	+	+	−	−	+	+	+	−	−			
Wycherley et al. (2010)	-0.2	0.6	21	0	0.5	19	26.1%	-0.20 [-0.54, 0.14]	+	+	−	−		+	+	−	+			
**Total (95% CI)**			**116**			**110**	**100.0%**	**-0.07 [-0.25, 0.10]**												

Heterogeneity: Tau² = 0.00; Chi² = 1.95, df = 3 (P = 0.58); I² = 0%
Test for overall effect: Z = 0.80 (P = 0.42)

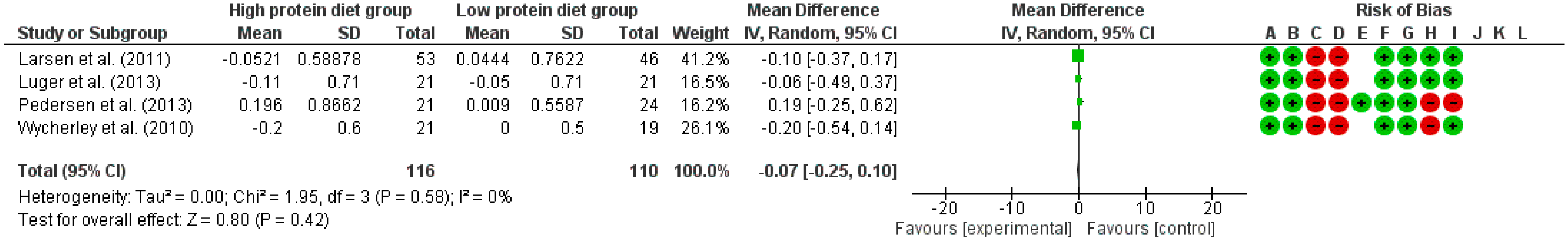

Risk of bias legend
(**A**) Clearly focused research question
(**B**) Random sequence generation (selection bias)
(**C**) Allocation concealment (selection bias)
(**D**) Blinding of participants and personnel (performance bias)
(**E**) Blinding of outcome assessment (detection bias)
(**F**) Groups are similar at the start
(**G**) Standardised, valid and reliable values
(**H**) Drop out rate
(**I**) Intention-to-treat analysis
(**J**) Incomplete outcome data (attrition bias)
(**K**) Selective reporting (reporting bias)
(**L**) Other bias

Figure 5: Results of the meta-analysis - Effect of high protein diet on low density lipoprotein in diabetes patients

1.6 Systolic blood pressure (mmHg)

Study or Subgroup	High protein diet group Mean	SD	Total	Low protein diet group Mean	SD	Total	Weight	Mean Difference IV, Random, 95% CI	A	B	C	D	E	F	G	H	I	J	K	L
Larsen et al. (2011)	-5.0259	11.4903	53	-0.7643	11.15458	46	60.4%	-4.26 [-8.73, 0.21]	+	+	−	−		+	+	+	+			
Luger et al. (2013)	-6.19	17.53	21	0.45	8.15	22	17.8%	-6.64 [-14.87, 1.59]	+	+	−	−		+	+	+	+			
Pedersen et al. (2013)	0	0	21	0	0	24		Not estimable	+	+	−	−	+	+	+	−	−			
Wycherley et al. (2010)	-16	13	21	-13	11	19	21.8%	-3.00 [-10.44, 4.44]	+	+	−	−		+	+	−	+			
**Total (95% CI)**			**116**			**111**	**100.0%**	**-4.41 [-7.88, -0.94]**												

Heterogeneity: Tau² = 0.00; Chi² = 0.42, df = 2 (P = 0.81); I² = 0%
Test for overall effect: Z = 2.49 (P = 0.01)

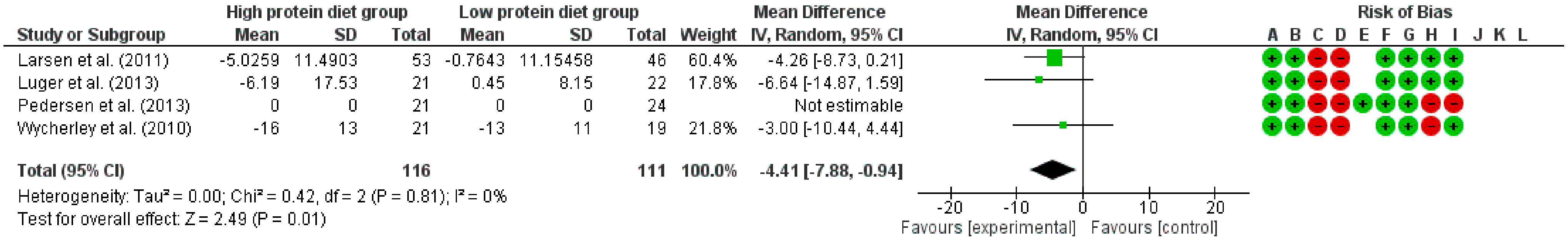

Risk of bias legend
(A) Clearly focused research question
(B) Random sequence generation (selection bias)
(C) Allocation concealment (selection bias)
(D) Blinding of participants and personnel (performance bias)
(E) Blinding of outcome assessment (detection bias)
(F) Groups are similar at the start
(G) Standardised, valid and reliable values
(H) Drop out rate
(I) Intention-to-treat analysis
(J) Incomplete outcome data (attrition bias)
(K) Selective reporting (reporting bias)
(L) Other bias

Figure 6: Results of the meta-analysis - Effect of high protein diet on systolic blood pressure in diabetes patients

1.7 Diastolic blood pressure (mmHg)

Figure 7: Results of the meta-analysis - Effect of high protein diet on diastolic blood pressure in diabetes patients

Larsen et al. (2011)	0.2052	11.24745	53	0.6499	11.3026	46	25.8%	-0.44 [-4.90, 4.01]
Luger et al. (2013)	-6.67	9.66	21	-1.59	10.28	22	16.4%	-5.08 [-11.04, 0.88]
Pedersen et al. (2013)	-2.54	6.37	21	4.23	9.25	24	24.7%	-6.77 [-11.37, -2.17]
Wycherley et al. (2010)	-10	6	21	-7	6	19	33.0%	-3.00 [-6.72, 0.72]
**Total (95% CI)**			**116**			**111**	**100.0%**	**-3.61 [-6.27, -0.95]**

Heterogeneity: Tau² = 1.98; Chi² = 4.09, df = 3 (P = 0.25); I² = 27%

Test for overall effect: Z = 2.66 (P = 0.008)

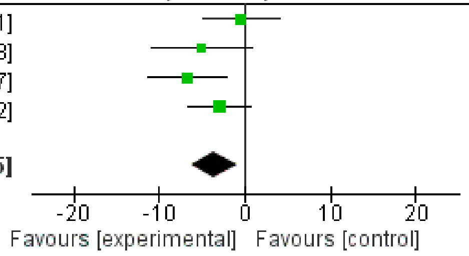

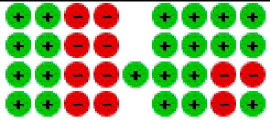

Risk of bias legend

(**A**) Clearly focused research question

(**B**) Random sequence generation (selection bias)

(**C**) Allocation concealment (selection bias)

(**D**) Blinding of participants and personnel (performance bias)

(**E**) Blinding of outcome assessment (detection bias)

(**F**) Groups are similar at the start

(**G**) Standardised, valid and reliable values

(**H**) Drop out rate

(**I**) Intention-to-treat analysis

(**J**) Incomplete outcome data (attrition bias)

(**K**) Selective reporting (reporting bias)

(**L**) Other bias

2 Meta-analysis - Low protein diets in diabetic nephropathy

2.1 Glomerular filtration rate (ml/min/1,73m²)

Figure 8: Results of the meta-analysis (Data were adapted and taken from Nezu et al.[1]) - Effect of low protein diet on GFR in diabetic nephropathy

Study or Subgroup	Mean	SD	Total	Mean	SD	Total	Weight	IV, Random, 95% CI	A	B	C	D	E	F	G	H	I	J	K	L
Dullaart 1993	-18	12.2	14	-10	6	16	11.0%	-8.00 [-15.03, -0.97]	+	+	−	−	−	−	+	+	+	−		
Dussol 2005	-7	11	22	-5	15	25	10.7%	-2.00 [-9.46, 5.46]	+	+	−	−	−	−	+	+	+			
Hansen 2002	-3.8	3.2	38	-3.9	3.9	38	13.9%	0.10 [-1.50, 1.70]	+	+		−	−	+	+	−	+			
Koya 2009	-21.4	22.8	56	-20.3	20	56	10.4%	-1.10 [-9.04, 6.84]	+	+	−	−	−	+	+	−	+	−		
Pijls 2002	-11.6	29	63	-14.9	32.7	68	8.6%	3.30 [-7.27, 13.87]	+	+	+	+	+	+	+	+	+	−		
Raal 1994	3	6.5	11	-8	6.4	11	12.1%	11.00 [5.61, 16.39]	+	+	−	−	+	+	+	+	−			
Velázquez 2008, macroalbuminuria	17.9	14.1	10	-9.3	7.5	9	9.0%	27.20 [17.18, 37.22]	+	+	−	−	−	+	+	+	−			
Velázquez 2008, microalbuminuria	6.5	9	10	-7.3	5.3	12	11.5%	13.80 [7.47, 20.13]	+	+	−	−	−	+	+	+	−			
Velázquez 2008, normoalbuminuria	-1.3	5.1	9	-2.9	3.5	10	12.9%	1.60 [-2.38, 5.58]	+	+	−	−	−	+	+	+	−			
**Total (95% CI)**			**233**			**245**	**100.0%**	**4.65 [-0.32, 9.62]**												

Heterogeneity: Tau² = 45.61; Chi² = 62.97, df = 8 (P < 0.00001); I² = 87%
Test for overall effect: Z = 1.83 (P = 0.07)

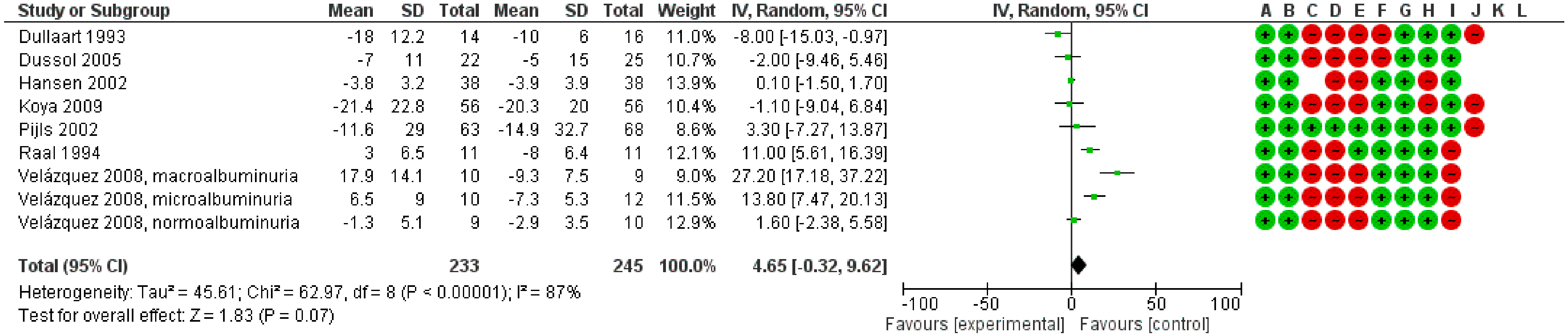

Risk of bias legend
(A) Clearly focused research question
(B) Random sequence generation (selection bias)
(C) Allocation concealment (selection bias)
(D) Blinding of participants and personnel (performance bias)
(E) Blinding of outcome assessment (detection bias)
(F) Groups are similar at the start
(G) Standardised, valid and reliable values
(H) Dropout rate
(I) Intention-to-treat analysis
(J) Incomplete outcome data (attrition bias)
(K) Selective reporting (reporting bias)
(L) Other bias

2.2 Proteinuria

Study or Subgroup	Experimental Mean	Experimental SD	Experimental Total	Control Mean	Control SD	Control Total	Weight	Std. Mean Difference IV, Random, 95% CI
Brinkworth 2004	0.2	0.1	19	-0.1	0.1	19	8.5%	2.94 [1.99, 3.88]
Dullaart 1993	-6	8.9	14	-2	2.2	16	9.4%	-0.62 [-1.36, 0.12]
Dussol 2005, macroalbuminuria	170	839.6	3	210	615.2	3	5.6%	-0.04 [-1.64, 1.56]
Dussol 2005, microalbuminuria	156	486	19	114	364	22	10.0%	0.10 [-0.52, 0.71]
Hansen 2002	-139	300	38	-107	612.2	34	10.6%	-0.07 [-0.53, 0.40]
Koya 2009	0.3	1.1	47	-0.5	1.1	41	10.7%	0.72 [0.29, 1.15]
Pijls 2002	1.2	23.5	63	-20.5	18.1	68	10.9%	1.03 [0.67, 1.40]
Raal 1994	-1	5.7	11	0.34	4.3	11	9.0%	-0.26 [-1.10, 0.58]
Velázquez 2008, macroalbuminuria	-536.3	868.1	10	-233	987.9	9	8.6%	-0.31 [-1.22, 0.59]
Velázquez 2008, microalbuminuria	1.4	34.6	10	34.12	57.4	12	8.8%	-0.65 [-1.51, 0.22]
Velázquez 2008, normoalbuminuria	3.5	4.8	9	-3.5	3.1	10	7.8%	1.68 [0.59, 2.76]
**Total (95% CI)**			**243**			**245**	**100.0%**	**0.40 [-0.13, 0.94]**

Heterogeneity: Tau² = 0.64; Chi² = 68.98, df = 10 (P < 0.00001); I² = 86%
Test for overall effect: Z = 1.49 (P = 0.14)

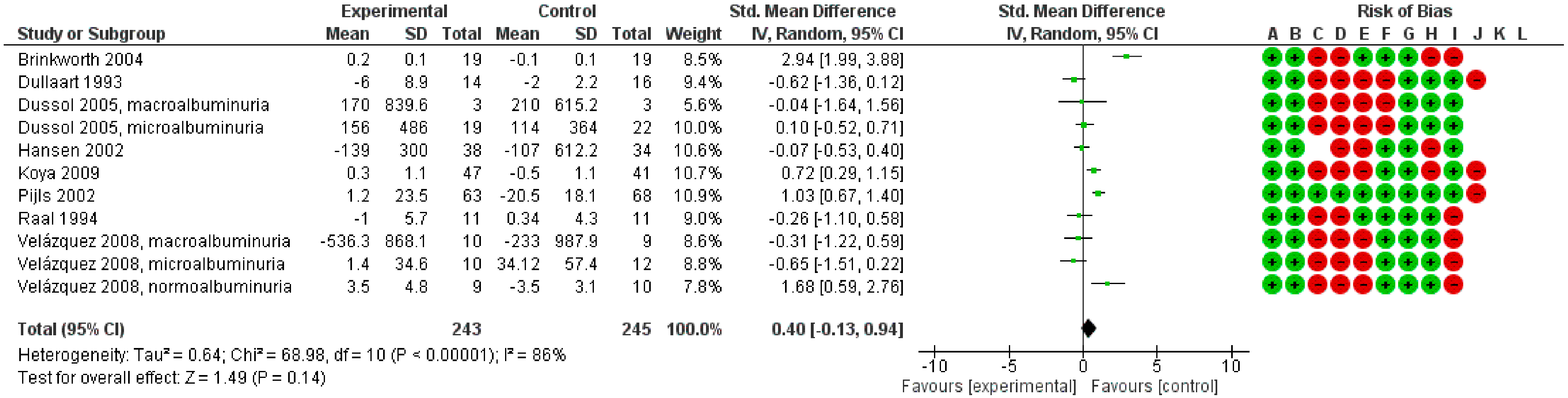

Study or Subgroup	A	B	C	D	E	F	G	H	I	J	K	L
Brinkworth 2004	+	+	−	−	+	+	+	−	−			
Dullaart 1993	+	+	−	−	−	−	+	+	+	−		
Dussol 2005, macroalbuminuria	+	+	−	−	−	−	+	+	+			
Dussol 2005, microalbuminuria	+	+	−	−	−	−	+	+	+			
Hansen 2002	+	+		−	−	+	+	−	+			
Koya 2009	+	+	−	−	−	+	+	−	+	−		
Pijls 2002	+	+	+	+	+	+	+	+	+	−		
Raal 1994	+	+	−	−	+	+	+	+	−			
Velázquez 2008, macroalbuminuria	+	+	−	−	−	+	+	+	−			
Velázquez 2008, microalbuminuria	+	+	−	−	−	+	+	+	−			
Velázquez 2008, normoalbuminuria	+	+	−	−	−	+	+	+	−			

Risk of bias legend
(**A**) Clearly focused research question
(**B**) Random sequence generation (selection bias)
(**C**) Allocation concealment (selection bias)
(**D**) Blinding of participants and personnel (performance bias)
(**E**) Blinding of outcome assessment (detection bias)
(**F**) Groups are similar at the start
(**G**) Standardised, valid and reliable values
(**H**) Dropout rate
(**I**) Intention-to-treat analysis
(**J**) Incomplete outcome data (attrition bias)
(**K**) Selective reporting (reporting bias)
(**L**) Other bias

Figure 9: Results of the meta-analysis (Data were adapted and taken from Nezu et al. [1]) - Effect of low protein diet on proteinuria in diabetic nephropathy

3 Tables of evidence

3.1 High protein diet in patients with type 2 diabetes mellitus (RCTs)

Author, year	Quality of evidence	SIGN	Study-type	Participants	Intervention				Results
					Duration	Ratio (carb:pro:fat)			
						prescribed (Interv./Control)	Achieved (Interv./Control)	Energy-R	
**Brinkworth, 2004 [2]**	1-	Acceptable (+)	RCT	38 T2DM	64 w	40:30:30 vs. 55:15:30	yes	yes	**weight:** -3,7±1 kg (HP) vs. -2,2±1,1 kg (LP); **HDL:** significant increase in both groups
**Jesudason, 2013 [3] Pedersen, 2013 [4]**	1 -	Acceptable (+)	RCT	76 T2DM	1 y	40:30:30 vs. 50:20:30	36:29:29 vs. 46:19:28	yes	**weight**: I: -9,7 ± 13,29; C: -6,6 ± 6,86 kg<br>**Fasting blood glucose**: I: -1,0 ± 1,375; C: -1,5 ± 2,45
**Larsen, 2011 [5]**	1 -	Acceptable (+)	RCT	99 T2DM	1 y (3 mo E-R + 9 mo E-B)	40:30:30 vs. 55:15:30	**0**: 44:21:32 vs. 45:20:33<br>**3 m**: 40:28:30 vs. 49:21:29<br>**12 m**: 42:26:31 vs. 48:19:32	yes	**weight**: I: -2.23 ± 3.78; C: -2.16 ± 4.26 kg **n.s.**<br>**HbA1c**: I: -0.23 ± 1.06; C: -0.28 ± 0.99 %<br>**HDL**: I: 0.08 ± 0.29; C: 0.07 ± 0.23 mmol/l<br>**LDL**: I: -0.05 ± 0.59; C: 0.04 ± 0.76 mmol/l<br>**SBP**: I: -5.03 ± 11.49; C: -0.76 ± 11.15 mmHg<br>**DBP**: I: 0.205 ± 11.25; C: 0.65 ± 11.30 mmHg
**Luger, 2013 [6]**	1 -	Acceptable	RCT	44 T2DM	12 w	40:30:30 vs. 55:15:30	**0:** 43:23:32 vs. 43:20:32<br>**4 w:** 37:26:34 vs. 48:17:30<br>**12 w:** 37:25:35 vs. 50:17:30	yes	
**Wycherley, 2010 [7]**	1 -	Acceptable (+)	RCT	83 T2DM	16 w	43:33:22 vs. 53:19:26	**16 w**: 47:32:18 vs. 54:19:23	yes	**weight**: I: -9.0 ± 4.8; C: -8.6 ± 4.6 kg<br>**HbA1c**: I: -1.8 ± 1.6; C: -1.1 ± 0.6 %<br>**Fasting BG**: I: -2.5 ± 2.7; C: -2.2 ± 2.2 mmol/l<br>**HDL**: I: -0.1 ± 0.2; C: 0 ± 0.2 mmol/l<br>**LDL**: I: -0.2 ± 0.6; C: -0.3 ±0.5 mmol/l<br>**SBP**: I: -16 ± 13; C: -13 ± 11 mmHg<br>**DBP**: I: -10 ± 6; C: -7 ± 6 mmHg

3.2 Overview of nutrient intake in high protein diet

Study	Year	N	Ratio	Duration		Point of time	Protein intake

			Carb:**Protein:**Fat		**E-R**[1]		**High protein diet**			**Normal protein diet**		
							**g**	**%**	**kcal**	**g**	**%**	**kcal**
**Brinkworth [2]**	2004	38	40:**30**:30 vs. 55:**15**:30	8 W ER; 4 W EB 12 mo Follow-up	Yes	8 weeks	*120g**	30 %	1595	*60g**	15 %	1595
**Jesudason [3] Pedersen [4]**	2013	76	40:**30**:30 vs. 50:**20**:30	1 year	Yes	-	110g	28.4%	1377	97 g	25.1 %	1408
**Krebs [8]**	2012	419	40:**30**:30 vs. 55:**15**:30	2 year	Yes	6 months<br>12 months<br>24 months	95 g<br>91 g<br>87 g	22 %<br>21 %<br>21 %	1762<br>1728<br>1707	81 g<br>83 g<br>85 g	20 %<br>21%<br>20%	1623<br>1615<br>1689
**Larsen [5]**	2011	99	40:**30**:30 vs. 55:**15**:30	1 year	Yes	3 months<br>12 months	*108 g**<br>*105 g**	28,2 %<br>26.5 %	1536<br>1587	*75 g**<br>*74 g**	20,8 %<br>18,9 %	1436<br>1578
**Luger [6]**	2013	44	40:**30**:30 vs. 55:**15**:30	12 weeks	Yes	0<br>4 weeks<br>12 weeks	*75 g**<br>*81 g**<br>*81 g*	22,9 %<br>26,5 %<br>25,6 %	1318<br>1219<br>1272	*66 g**<br>*57g**<br>*51g**	19,8 %<br>17,0%<br>16,6 %	1326<br>1332<br>1235
**Luscombe [9]**	2002	26	42:**28**:30 vs. 55:**16**:29	8 + 4 weeks	Yes	8 weeks<br>+ 4 weeks	*112 g**<br>*128 g**	28,2 %<br>27,7 %	1585<br>1844	*64 g**<br>*70 g**	16,1 %<br>15,8 %	1583<br>1777
**Parker [10]**	2002	66	42:**28**:28 vs. 55:**16**:27	8 + 4 weeks	Yes	8 weeks<br>+ 4 weeks	*112 g**<br>*140 g**	28,1 %<br>27,7 %	1587<br>2029	*63 g**<br>*71 g**	16,4 %<br>16,0 %	1543<br>1785
**Wycherley [7]**	2010	28	47:**32**:18 vs. 53:**18**:22	16 weeks	Yes	-	119g	32,3%	1505	68g	18,6%	1494

** = estimated values*

[1] E-R = Energy restriction

3.3 Working document – Results of high protein diets in diabetes patients

**Author, Year**	**Study Design**	**Population**	**Intervention and Control** (carb:**pro**:fat)	**Outcome A**<br>***Weight loss (Mean ± SD)***	**Outcome B**<br>***HbA1c (Mean ± SD)***	**Outcome C**<br>***Fasting blood glucose (Mean ± SD)***	**Outcome D**<br>***HDL (Mean ± SD)***	**Outcome E**<br>***LDL (Mean ± SD)***	**Outcome F**<br>***SBP (Mean ± SD)***	**Outcome G**<br>***DBP (Mean ± SD)***
**Brinkworth, 2004 [2]**	RCT	Type 2 DM<br>I: 19<br>C: 19	I: 40:**30**:30<br>C: 55:**15**:30	I: -3.7 ± 4.36<br>C: -2.2 ± 4.79	-	-	-	-	-	-
**Larsen, 2011 [5]**	RCT	Type 2 DM<br>I: 53<br>C:46	I: 40:**30**:30<br>C: 55:**15**:30	*I: -2.23 ± 3.78*<br>C: -2.16 ± 4.26**	*I: -0.23 ± 1.06*<br>C: -0.28 ± 0.99**	-	*I: 0.08 ± 0.29*<br>C: 0.07 ± 0.23**	*I: -0.05 ± 0.59*<br>C: 0.04 ± 0.76**	*I: -5.03 ± 11.49*<br>C: -0.76 ± 11.15**	*I: 0.205 ± 11.25*<br>C: 0.65 ± 11.30**
**Luger, 2013[6]**	RCT	Type 2 DM<br>I: 21<br>C: 22	I: 40:**30**:30<br>C: 55:**15**:30	*I: -3.09 ± 2.44*<br>C: -1.01 ± 0.05**	*I: -0.25 ± 0.56*<br>C: -0.19 ± 0.60**	*I: -2.32 ± 3.47*<br>C: -0.12 ± 2.17**	*I: 0.02 ± 0.18*<br>C: 0.04 ± 0.15**	*I: -0.11 ± 0.71*<br>C: -0.05 ± 0.71**	*I: -6.19 ± 17.53*<br>C: 0.45 ± 8.15**	*I: -6.67 ± 9.66*<br>C: -1.59 ± 10.28**
**Pedersen, 2013 [4]**	RCT	Type 2 DM<br>I: 21<br>C:24	I: 40:**30**:30<br>C: 50:**20**:30	*I: -9.7 ± 13.36*<br>C: -6.6 ± 7.10**	*I: -0.352 ± 1.12*<br>C: -0.25 ± 0.8**	*I: -1.0 ± 1.375*<br>C: -1.5 ± 2.45**	*I: 0.49 ± 2.10*<br>C: 0.08 ± 0.23**	*I: 0.19 ± 0.86*<br>C: 0.009 ± 0.55**	*I: -4.79 ± 13.08*<br>C: 1.79 ± 11.19**	*I: 2.54 ± 6.37*<br>C: 4.23 ± 9.25**
**Wycherley, 2010 [7]**	RCT	Type 2 DM<br>I: 21<br>C:19	I: 47:**32**:18<br>C: 53:**18**:22	I: -9.0 ± 4.8<br>C: -8.6 ± 4.6	I: -1.8 ± 1.6<br>C: -1.1 ± 0.6	I: -2.5 ± 2.7<br>C: -2.2 ± 2.2	I: -0.1 ± 0.2<br>C: 0 ± 0.2	I: -0.2 ± 0.6<br>C: -0.3 ±0.5	I: -16 ± 13<br>C: -13 ± 11	I: -10 ± 6<br>C: -7 ± 6

* = significant values

*Cursive values = requested from the authors**

3.4 Excluded RCTs

Author, year	Study-type	Participants	Intervention		Exclusion
			Duration	Ratio (Carb:Protein:Fat)	
**Bibra, 2013 [11]**	Crossover	16 T2DM	2+3 w	25:30:45 vs. 55:20:25	difference between fat intake too high
**Boden, 2005 [12]**	CT	10 T2DM	2 w	Data in g per day:<br>day 1 – 7: CHO: 309 g; F: 154 g; P: 137 g<br>day 8 – 21: CHO: 21 g; F: 4 g; P: 151 g	no randomization, high risk of bias,<br>difference between protein intake too little
**Daly, 2006 [13]**	RCT	102 T2DM	12 w	34:26:40 vs. 46:20:33	difference between fat intake too high
**Davis, 2011 [14]**	RCT	27 T2DM	6 mo	Low Carb vs. Low Fat	no difference in protein intake
**Davis, 2009 [15]**	RCT	105 T2DM	1 y	24:27:49 vs. 53:22:25	difference between fat intake too high
**De Mello, 2011 [16]**	RCT	104 P with impaired glucose tolerance	12 w	-	non-diabetics, no information about protein intake
**Dyson, 2008 [17]**	Review	521 T2DM	-	-	
**Dyson, 2007 [18]**	RCT	26 (13 diabetic, 13 non-diabetics)	3 mo	17:31:46 vs. 39:20:34	too little diabetics, difference of fat intake too high
**Elhayany, 2010 [19]**	RCT	259 diabetics	12 mo	LC mediter. diet: 35:20:45<br>traditional mediter. diet: 50:20:30<br>ADA nutrition: 50:20:30	comparison mediterranean diet, difference fat intake, no difference between protein intake
**Gannon, 2004 [20]**	Crossover	8 T2DM	5 w	20:30:50 vs. 55:15:30	fat intake too high
**Gutierrez, 1998 [21]**	CT	28 T2DM	8 + 12 w	25:45:30 vs. 55:20:25	low quality, difference between fat intake too high
**Hussain, 2012 [22]**	RCT	363 obese (102 T2DM)	24 w	-	no data about nutrition
**Keogh, 2007 [23]**	RCT	73 obese hyperinsulinemic	12 mo	30:20:50 vs. 30:40:30	non-diabetics
**Kirk, 2008 [24]**	Metaanalyse	T2DM	-	-	difference of protein intake?
**Khoo, 2011 [25]**	RCT	31 T2DM	8w + 44w	0,8 g Protein/kg [LP] vs. 300 g lean meat [HP]	high risk of bias, very high dropout-rate (ca. 50%)
**McAuley, 2006 [26]**	RCT	93 insulinresistent women	12 mo	HP: 37:22:37; HF: 33:21:41<br>HC: 45:22:29	non-diabetics
**McAuley, 2005 [27]**	RCT	93 insulinresistent women	24 w	HP: 35:26:35; HF: 26:24:47<br>HC: 45:21:28	non-diabetics
**McCarthy, 2012 [28]**	Review	-	-	-	non-diabetics
**Navas-Carretero, 2011 [29]**	Longitudinal Studie	17 T2DM	4w+4w	-	no RCT, difference of fat intake
**Papakonstantinou, 2010 [30]**	Crossover	17 T2DM	4 w	50:30:20 vs. 50:15:35	difference of fat intake too high
**Samaha, 2003 [31]**	RCT	132 obese (39% diabetic)	6 mo	LF: 51:16:33	too little diabetics, difference of fat intake too high

				LC: 37:22:41	
**Seshadri, 2004 [32]**	RCT	78 obese (31 T2DM, 36 MetS)	6 mo	LC: CHO =32 ± 20% ; P=25±9%; F=43±17%<br>Conventional Diet: CHO=50±16%; P=16±5%; F=33±14%	difference of fat intake too high, difference of protein intake?
**Stern, 2004 [33]**	RCT	132 obese<br>(82 T2DM)	1 y	CHO<30g vs. caloric reduction of 500 kcal	fat intake different, not only diabetics
**Tay, 2014 [34]**	RCT	115	24 w	14:28:58 vs. 53:17:30	difference of fat intake too high
**Westman, 2008 [35]**	RCT	50 T2DM	24 w	13:28:59 vs. 44:20:36	difference of fat intake too high
**Wheeler, 2012 [36]**	Systematic Review	T2DM	-	-	
**Wolever, 2008 [37]**	RCT	162 T2DM	1 y	High GI: 47:22:31<br>Low GI: 52:21:27<br>LC: 39:21:40	difference of fat intake too high<br>no difference between protein intake
**Yancy, 2010 [38]**		146 participants<br>31% diabetics	1 y	34:32:34 vs. 62:25:13	difference of fat intake too high

3.5 Different types of protein in patients with type 2 diabetes mellitus (RCT)

Author, year	Quality of evidence	SIGN	Study-type	Participants	Intervention		Results			
					Duration	Intervention vs. control	Glycaemic control/HbA1c	Proteinuria/GFR	Serumlipid	Bloodpressure
**Azadbakht, 2008 [39]**	1-	Acceptable (+)	RCT	41 T2DM with nephropathy	4 y	**Soy protein** (0,8 g/kg/d; 35% animal,35% TVP, 30% vegetable) vs. **Control** (0,8 g/kg/d; 70% animal, 30% vegetable)	FPG<br>SP: 141+-55 >121+-42<br>CG: 137+-54>147+-57<br>T*G P=0.02	**SP:**<br>**84+-19 > 88+-33**<br>**CG:**<br>**78+-23 > 81 +-35**<br>**T*G n.s.**	**TC: SP:**<br>**225+-48 > 201+-35**<br>**CG:**<br>**218+-38 > 228 +- 48**<br>**T*G p=0.01**<br>**TG, LDL, HDL n.s.**	n.s.
**Pecis, 1994 [40]**	1-	Acceptable (+)	RCT, crossover	15 T1DM	13 w (3x3 w interv. and 2 w washout)	**Usual diet** (meat: 79,4% beef, 20,6% chicken) vs. **Low protein diet** (0,5g/kg/d vegetable and milk protein, 7% P/60% CHO/33% F) vs. **Test diet** (same like usual, red meat replaced by 85% chicken and 15% fish)	No change	**GFR:** significant ↓ after LPD and test diet<br><br>**UAE:** no change	**Chol:** significant ↑ in usual diet<br>**HDL:** no change<br>**TG:** no change	No change
**Wheeler, 2002 [41]**	1-	Acceptable (+)	RCT, crossover	23 T2DM	16 w (2x6 w interv. and 4 w washout)	**Animal protein** (60% animal, 40% plant) vs. **Plant protein** (tofu, TVP, soy, legumes)	**AP:** 7,9%→7,4% (P<0,01)<br>**PP:** 8,1%→7,5% (P<0,01)<br>**No diet effect**	**GFR and AER**: no change	**Chol:** 4,75→4,34 mmol/l (P<0,01) in both groups<br>**TG:** no change<br>**HDL:** no change	**Diastolic pressure:**<br>**AP:**82→78 mmHg (P<0,02)<br>**PP:**83→80 mmHg (P<0,02)<br>**Systolic pressure:**<br>no change

**Gross, 2002 [42]**	1-	Acceptable (+)	RCT, crossover	33 T2DM	20 w (3x4 w interv. and 4 w washout)	**Usual diet** (*achieved 1,43±0,3 g/kg/d*) vs. **Low protein** (0,5-0,8 g/kg/d, only milk and vegetable protein, *achieved 0,66±0,2 g/kg/d*) vs. **Chicken diet** (1,2-1,5 g/kg/d, red meat replaced by chicken*, achieved 1,35±0,3 g/kg/d*)	No change	**GFR in normoalbuminuric:** Lower after chicken (101,3±22,9 ml/min/1,73m^2) and low protein (93,8±20,5ml/min/1,73m^2) than after usual (113,4±31,4ml/min/1,73m^2), P<0,05<br><br>**GFR in microalbuminuric:** Lower after low protein (93,5±8,5ml/min/1,73m^2) than after chicken (102,8±22,5ml/min/1,73m^2) and usual (107,1±20,1ml/min/1,73m^2), P<0,05<br><br>**UAER in normoalbuminuric:** No change<br><br>**UAER in microalbuminuric:** After chicken (median 34,3µg/min) significantly lower than after usual (median 63,8µg/min) and low protein (median 52,3µg/min), P<0,05	**Normoalbuminuric:** No change in chol, HDL, LDL, apolipo B, TG<br><br>**Microalbuminuric:** Apolipo B significantly lower after chicken(113,5±36mg/dl) and low protein (103,5±40,1mg/dl) than after usual (134,3±30,7mg/dl), P<0,05	No change
**Pipe, 2009 [43]**	1-	Acceptable (+)	RCT, crossover, doubleblind, placebo-controlled	34 T2DM	2x57 d with 28 d washout	**Soy protein isolate** (40 g protein and 88 mg isoflavones) vs. **Milk protein isolate** (40 g protein, no isoflavones)	Not measured	Not measured	**LDL:**<br>**MPI:** 2,98±0,14→2,9±0,12mmol/l<br>**SPI:** 2,95±0,12→2,78±0,13mmol/l P=0,04<br><br>**LDL:HDL:**<br>**MPI:** 2,66±0,12→2,66±0,11<br>**SPI:** 2,53±0,1→2,5±0,1	Not measured

									P=0,02<br><br>**Apolipo B:apolipo A-I:**<br>**MPI:** 0,67±0,03→0,67±0,03<br>**SPI:** 0,67±0,03→0,64±0,03<br>P=0,05	

3.6 Low protein intake in diabetic nephropathy (RCT)

Author, year	Quality of evidence	SIGN	Study-type	Participants	Intervention					Results		
					Duration	Protein intake (g/kg Bodyweight)<br>"Low protein" "Normal protein"						
						prescribed	achieved	prescribed	achieved	Nephro pathy	GFR	HbA1c
**Dullaart, 1993 [44]**	1 -	Accep table (+)	RCT	31 IDDM	2 y	0,6	0,79	free	1,09	Microalb.	GFR-changes:<br>LP: 131 ± 34 → 120 ± 20 → 113 ± 24<br>NP: 122 ± 26 → 119 ± 19 → 112 ± 21	changes :<br>LP: 7,84 ± 0,93 → 8,02 ± 0,85<br>NP: 7,82 ± 1,01 → 8,01 ± 1,20
**Dussol, 2005 [45]**	1-	Accep table (+)	RCT	63 T1DM + T2DM	24 mo	0,8	0,87	1,2	1,03	Microalb + Macroalb.	GFR- changes :<br>LP: 82 ± 21 → 80 ± 23 → 74 ± 25<br>decline: -7 ± 11<br>NP: 89 ± 27 → 84 ± 33 → 82 ± 24<br>decline:-5 ± 15	changes:<br>LP:8,4 ± 1,8 → 8,2 ± 1,3 → 7,9 ± 2,1<br>NP:8,0 ± 1,1 → 8,2 ± 1,4 →8,1 ± 2,2
**Hansen, 2002 [46]**	1 -	Accep table (+)	RCT	82 T1DM	4 y	0,6	0,89	free	1,02	Macroalb.	GFR-decline:<br>LP: 7,6 (4,9 – 10,2) → -3,8 (2,8 – 4,8)<br>NP:6,6 (5,2 – 8,1) → -3,9 (2,7 – 5,2)	changes :<br>LP: 9,8 % (9,4 – 10,1) → 9,5 (9,1 – 9,9)<br>NP: 9,6% (9,2 – 9,9) → 9,6 (9,3 – 10,0)
**Koya, 2009 [47]**	1 -	Accep table (+)	RCT	112 T2DM	60 mo	0,8	1,0	1,2	1,0	Macroalb.	eGFR (annual changes):<br>LP: - 6,1 ± 6,5<br>NP: -5,8 ± 5,7	Baseline:<br>LP: 7,8 ± 1,5<br>NP: 7,5 ± 1,7
**Meloni, 2002 [48]**	1 -	Accep table (+)	RCT	69 (32 T1 and 37 T2DM)	1 y	0,6	0,68 ± 0,21	free	1,39 ± 0,28	Diabet. Nephrop.	GFR- changes :<br>LP: 43 ± 4,7 →38 ± 9,6<br>NP: 45 ± 5,1 →39 ± 7,2	changes:<br>LP: 7,2 ± 0,5 →6,0 ± 1,1<br>NP: 6,7 ± 0,5 →6,2 ± 0,8
**Pedersen, 2013 [4]**			RCT	45 T2DM	1 y	-	-	-	-	Microalb. + Macroalb.	iGFR- changes :<br>HP: 108 ± 7,3 → 101 ± 6,1<br>NP: 91,9 ± 5,5 → 93,9 ± 5,4	changes:<br>HP: 7,5 ± 0,2 → 7,2 ± 0,2<br>NP: 7,2 ± 0,1 → 6,9 ± 0,2
**Pijls, 2002 [49]**	1 -	Accep table (+)	RCT	131 T2DM	28 ± 7 mo	0,8	1,1	free	1,14	Normoalb. + Microalb.	GFR decline<br>6 Mo- LP: -2,9 ± 17 ; NP: -1,3 ± 15	Baseline:<br>LP: 7,7 ± 1,4<br>NP: 7,7 ± 1,5

											12 Mo-LP: -4,8 ± 12; NP: -6,4 ± 14	
**Raal, 1994 [50]**	1 -	Accep table (+)	RCT	22 IDDM	6 mo	0,8	0,87	1,6	2,0	Macroalb.	GFR- changes :<br>LPD: 50 ± 19 → 53 ±23<br>UPD: 66 ± 28 → 58 ± 26	changes:<br>LPD: 12,0 ± 3,4 → 11,7 ± 4,6<br>UPD: 13,9 ± 2,4 → 12,4 ± 5,5
**Velazquez, 2008 [51]**	1 -	Accep table (+)	RCT	T2DM	4 mo	0,6 – 0,8	0,82	1,0 – 1,2	1,2	Normoalb. + Microalb + Macroalb.	GFR- changes :<br>LPD normo: 87,5 ± 15,2 →86,2 ± 18,2<br>LPD microalb: 69,7 ± 36,9 → 76,2 ± 35,6<br>LPD macroalb: 56,3 ± 29,0 → 74,2 ± 40,4<br>NPD normo: 81,5 ± 21,7 → 78,6 ± 19,7<br>NPD microalb: 89,2 ± 32,1 → 81,9 ± 34,6<br>NPD macroalb: 74,4 ± 31,4 → 65,1 ± 25,5	changes:<br>LPD normo: 7,5 ± 1,5 → 6,8 ± 0,8<br>LPD microalb: 8,2 ± 1,6 → 7,2 ± 1,8<br>LPD macroalb: 8,4 ± 2,1 → 7,6 ± 1,0<br>NPD normo: 8,8 ± 2,2 → 7,9 ± 1,3<br>NPD microalb: 8,8 ± 1,9 → 7,1 ± 0,8<br>NPD macroalb: 8,1 ± 1,8 → 6,9 ± 1,6
**Walker, 1989 [52]**	-	Reject (-)	CT	19 IDDM	NP: 29 mo<br>LP: 33 mo	-	0,67	-	1,13	Macroalb. Alb-excretion: > 300µg	GFR-decline:<br>LP: 0,14 ml/min per month<br>NP: 0,61 ml/min per month (signif.)	?
**Zeller, 1991 [53]**	-	Reject (-)	RCT	35 T1DM with Nephropathy	Ø 3 y	0,6	0,72	> 1,0	1,08	Macroalb.		7,9 %

3.7 Low protein intake in diabetic nephropathy (existing meta-analysis)

Author, Year	Evidence	SIGN	Study-type	Aim	Search strategy	Inclusion criteria	Participants	Results
**Kasiske, 1998 [54]**	0	Unacceptable - Reject	MA	Effect of low-protein diet on kidney function in diabetic nephropathy	Not described		23 studies (6 studies with diabetes patients and 17 persons without diabetes	Dietary protein restriction retards the rate of renal function decline.
**Maeda, 2007 [55]**	0	No SR	R	Diet therapy in diabetic nephropathy	No Systematic Review			Protein restriction should be prescribed for patients with diabetic nephropathy, as far as calorie intake is sufficient and the prescribed protein intake does not cause malnutrition.
**Nezu, 2013 [1]**	1 ++	High quality (++)	SR + MA	Effect of low-protein diet on kidney function in diabetic nephropathy	PubMed, EMBASE, Cochrane library, ClinicalTrials.gov, International Standard RCT, UMIN-CTR	Fulltext available<br>- RCT<br>- measured: GFR, CCr, proteinurie, albuminuria, HbA1c, serum albumin	779 Persons with Diabetes mellitus Type 1 and type 2	A diet intervention by a low protein diet hast modest but significant effects on the course of kidney prognosis in patients with diabetic nephropathy, especially when the intervention is sustainable regarding patients compliance.<br><br>The quality of the evidence for GFR was low.
**Otoda, 2014 [56]**	0	No SR	R	Protein restriction in diabetic nephropathy	MEDLINE, PubMed, EMBASE, ClinicalTrials.gov, Cochrane Controlled Clinical Trials	No Systematic Review		The significant benefits of LPD on progressive renal diseases in rodent and human studies did not reveal that there is much impact of the renoprotective strategies against kidney disease including diabetes.

**Pan, 2008 [57]**	1 +	Acceptab le (+)	MA	Protein restriction in diabetic nephropathy	MEDLINE, EMBASE, ClinicalTrials.gov, Cochrane Controlled Clinical Trials	- > 6 month<br>- RCT<br>- measured: GFR, CCr)	8 included studies (Type 1 und Type 2 Diabetes with diabetic nephropathy)	A low protein diet was not associated with a significant improvement of renal function in patients with either type 1 and 2 diabetic nephropathy
**Pedrini, 1996 [58]**	0	Unaccept able - Reject	MA	The effect of dietary protein restriction on the progression of renal disease	MEDLINE, references in review articles	- Fulltext available<br>- RCT	5 studies with persons without diabetes (1413 participants)<br>5 studies with insulin dependent diabetes (108 participants)	Dietary protein restriction effectively slows the progression of both diabetic and nondiabetic renal disease
**Robertson, 2009 [59]**	1 ++	High quality (++)	SR	Protein restriction for diabetic renal disease	The Cochrane library<br>MEDLINE<br>EMBASE<br>ISI Proceedings<br>Science citation index expanded	- > 4 month<br>- Type 1 und type 2 diabetes<br>- Comparison Low-Protein vs. Normal (Usual-) Protein	160 persons with Diabetes mellitus Type 1 und type 2	Overall, a restricted protein intake does appear to slow the progression of diabetic nephropathy albeit in a non-significant way. Studies did not give sufficient details to quantify this.

3.8 Working document – Results of low protein diet in diabetic nephropathy

Author, Year	Study Type	Population	Actual Protein intake (g/kg/BW)	Outcome A<br><br>*GFR changes ml/min/1,73 m²*<br>*(Mean ± SD)*	Outcome B<br><br>*HbA1c %*<br>*(Mean ± SD)*
**Dullaart, 1993 [44]**	RCT	31 IDDM	I: 0,79<br>C: 1,09	Changes in GFR:<br>LP: 131 ± 34 → 120 ± 20 → 113 ± 24<br>NP: 122 ± 26 → 119 ± 19 → 112 ± 21	LP: 7,84 ± 0,93 → 8,02 ± 0,85<br>NP: 7,82 ± 1,01 → 8,01 ± 1,20
**Dussol, 2005 [45]**	RCT	63 T1+T2DM	I: 0,87<br>C: 1,03	Changes in GFR:<br>LP: 82 ± 21 → 80 ± 23 → 74 ± 25<br>decline: -7 ± 11<br>NP: 89 ± 27 → 84 ± 33 → 82 ± 24<br>decline:-5 ± 15	LP:8,4 ± 1,8 → 8,2 ± 1,3 → 7,9 ± 2,1<br>NP:8,0 ± 1,1 → 8,2 ± 1,4 →8,1 ± 2,2
**Hansen, 2002 [46]**	RCT	82 T1DM	I: 0,89<br>C: 1,02	Decline of GFR:<br>LP: 7,6 (4,9 – 10,2) → - 3,8 (2,8 – 4,8)<br>NP:6,6 (5,2 – 8,1) → -3,9 (2,7 – 5,2)	LP: 9,8 % (9,4 – 10,1) → 9,5 (9,1 – 9,9)<br>NP: 9,6% (9,2 – 9,9) → 9,6 (9,3 – 10,0)
**Koya, 2009 [47]**	RCT	112 T2DM	I: 1,0<br>C: 1,0	eGFR (annual changes):<br>LP: - 6,1 ± 6,5<br>NP: -5,8 ± 5,7	-
**Meloni, 2002 [48]**	RCT	69 T1+T2DM	I: 0,68<br>C: 1,39	Changes in GFR:<br>LP: 43 ± 4,7 →38 ± 9,6<br>NP: 45 ± 5,1 →39 ± 7,2	LP: 7,2 ± 0,5 →6,0 ± 1,1<br>NP: 6,7 ± 0,5 →6,2 ± 0,8
**Pedersen, 2013 [4]**	RCT	45 T2DM	-	Changes in iGFR:<br>HP: 108 ± 7,3 → 101 ± 6,1<br>NP: 91,9 ± 5,5 → 93,9 ± 5,4	LP: 7,2 ± 0,5 →6,0 ± 1,1<br>NP: 6,7 ± 0,5 →6,2 ± 0,8
**Pijls, 2002 [49]**	RCT	131 T2DM	I: 1,1<br>C: 1,14	Decline of GFR<br>6 mo- LP: -2,9 ± 17 ; NP: -1,3 ± 15<br>12 mo-LP: -4,8 ± 12; NP: -6,4 ± 14	-
**Raal, 1994 [50]**	RCT	22 IDDM	I: 0,87<br>C: 2,0	Changes in GFR:<br>LPD: 50 ± 19 → 53 ±23<br>UPD: 66 ± 28 → 58 ± 26	LPD: 12,0 ± 3,4 → 11,7 ± 4,6<br>UPD: 13,9 ± 2,4 → 12,4 ± 5,5
**Velazquez, 2008 [51]**	RCT	60 T2DM	I: 0,82<br>C: 1,2	Changes in GFR:<br>LPD normo: 87,5 ± 15,2 →86,2 ± 18,2<br>LPD microalb: 69,7 ± 36,9 → 76,2 ± 35,6<br>LPD macroalb: 56,3 ± 29,0 → 74,2 ± 40,4<br>NPD normo: 81,5 ± 21,7 → 78,6 ± 19,7<br>NPD microalb: 89,2 ± 32,1 → 81,9 ± 34,6<br>NPD macroalb: 74,4 ± 31,4 → 65,1 ± 25,5	LPD normo: 7,5 ± 1,5 → 6,8 ± 0,8<br>LPD microalb: 8,2 ± 1,6 → 7,2 ± 1,8<br>LPD macroalb: 8,4 ± 2,1 → 7,6 ± 1,0<br>NPD normo: 8,8 ± 2,2 → 7,9 ± 1,3<br>NPD microalb: 8,8 ± 1,9 → 7,1 ± 0,8<br>NPD macroalb: 8,1 ± 1,8 → 6,9 ± 1,6

4 GRADE

4.1 Summary of findings table - high protein diets in diabetes patients

high protein diet compared to normal protein diet for diabetes mellitus

Patient or population: patients with diabetes mellitus
Settings: outpatient
Intervention: high protein diet
Comparison: normal protein diet

Outcomes	Illustrative comparative risks* (95% CI)		Relative effect (95% CI)	No of Participants (studies)	Quality of the evidence (GRADE)	Comments
	Assumed risk<br>**Normal protein diet**	Corresponding risk<br>**High protein diet**				
**Weight loss (kg)**<br>Scale<br>Follow-up: 4 to 15 months	The mean weight loss (kg) ranged across control groups from<br>**-1.01 to 8.6 kg**	The mean weight loss (kg) in the intervention groups was<br>**1.21 lower**<br>(2.17 to 0.24 lower)		265<br>(5 studies)	⊕⊕⊖⊖<br>**low**[1,2,3,4,5,6]	
**Fasting blood glucose (mmol/l)**<br>Standard methods<br>Follow-up: 3 to 12 months	The mean fasting blood glucose (mmol/l) ranged across control groups from<br>**-0.12 to -1.5 mmol/l**	The mean fasting blood glucose (mmol/l) in the intervention groups was<br>**0.72 lower**<br>(1.98 lower to 0.54 higher)		128<br>(3 studies)	⊕⊖⊖⊖<br>**very low**[1,2,5,7,8,9,10]	
**HbA1c (%)**<br>High performance liquid chromatography (HPLC)<br>Follow-up: 3 to 12 months	The mean hba1c (%) ranged across control groups from<br>**-0.19 to -1.1 %**	The mean hba1c (%) in the intervention groups was<br>**0.1 lower**<br>(0.33 lower to 0.14 higher)		227<br>(4 studies)	⊕⊕⊖⊖<br>**low**[1,2,5,6,7,8]	
**High density lipoprotein (HDL) (mmol/l)**<br>Standard methods<br>Follow-up: 3 to 12 months	The mean high density lipoprotein (hdl) (mmol/l) ranged across control groups from<br>**0 to 0.083 mmol/l**	The mean high density lipoprotein (hdl) (mmol/l) in the intervention groups was<br>**0.02 lower**<br>(0.07 lower to 0.04 higher)		226<br>(4 studies)	⊕⊕⊖⊖<br>**low**[1,2,5,6,7,8]	
**Low density lipoprotein (LDL) (mmol/l)**<br>Standard methods<br>Follow-up: 3 to 12 months	The mean low density lipoprotein (ldl) (mmol/l) ranged across control groups from<br>**-0.05 to 0.0444 mmol/l**	The mean low density lipoprotein (ldl) (mmol/l) in the intervention groups was<br>**0.07 lower**<br>(0.25 lower to 0.1 higher)		226<br>(4 studies)	⊕⊕⊖⊖<br>**low**[1,2,5,6,7,8]	
**Systolic blood pressure (mmHg)**<br>Automated sphygmanometer<br>Follow-up: 3 to 12 months	The mean systolic blood pressure (mmhg) ranged across control groups from<br>**-13 to 0.45 mmHg**	The mean systolic blood pressure (mmhg) in the intervention groups was<br>**4.41 lower**<br>(7.88 to 0.94 lower)		227<br>(4 studies)	⊕⊕⊖⊖<br>**low**[1,2,5,6,7,8]	

**Diastolic blood pressure**<br>Automated sphygmanometer<br>Follow-up: 3 to 12 months	The mean diastolic blood pressure ranged across control groups from<br>**-7 to 4.23 mmHg**	The mean diastolic blood pressure in the intervention groups was<br>**3.61 lower**<br>(6.27 to 0.95 lower)	227<br>(4 studies)	⊕⊕⊖⊖<br>**low**[1,2,5,6,7,8]

*The basis for the **assumed risk** (e.g. the median control group risk across studies) is provided in footnotes. The **corresponding risk** (and its 95% confidence interval) is based on the assumed risk in the comparison group and the **relative effect** of the intervention (and its 95% CI).

CI: Confidence interval;

GRADE Working Group grades of evidence
High quality: Further research is very unlikely to change our confidence in the estimate of effect.
Moderate quality: Further research is likely to have an important impact on our confidence in the estimate of effect and may change the estimate.
Low quality: Further research is very likely to have an important impact on our confidence in the estimate of effect and is likely to change the estimate.
Very low quality: We are very uncertain about the estimate.

[1] The subjects were not blinded to treatment allocation
[2] With one exception there were no blinding of outcome assessment
[3] Two studies did not analyse all subjects in the groups which they were randomly allocated (intention-to-treat)
[4] Three studies had a high dropout rate. (> 20%)
[5] Publication bias was not assessed as there were inadequate numbers of included trials to properly assess a funnel plot or more advanced regression-based assessments
[6] Two studies were partly funded by the meat industry
[7] Two studies had a high dropout rate (> 20%)
[8] One study did not analyses all subjects in the groups which they were randomly allocated (intention-to-treat)
[9] I^2 = 54% (Cochrane Handbook 5.0: substantial heterogeneity)
[10] One study were partly funded by the meat industry

4.2 GRADE profile – high protein diets in diabetes patients

Question: Should high protein diet vs normal protein diet be used for diabetes mellitus?

Bibliography: High protein diets for Type 2 diabetes

Quality assessment							Summary of Findings				
**Participants (studies) Follow up**	**Risk of bias**	**Inconsistency**	**Indirectness**	**Imprecision**	**Publication bias**	**Overall quality of evidence**	**Study event rates (%)**		**Relative effect** (95% CI)	**Anticipated absolute effects**	
							**With Normal protein diet**	**With High protein diet**		**Risk with Normal protein diet**	**Risk difference with High protein diet** (95% CI)
**Weight loss (kg)** (IMPORTANT OUTCOME; measured with: Scale; Better indicated by lower values)											
265 (5 studies) 4 to 15 months	very serious[1,2,3,4]	no serious inconsistency	no serious indirectness	no serious imprecision	undetected[5,6]	⊕⊕⊖⊖ **LOW**[1,2,3,4,5,6] due to risk of bias	130	135	-	The mean weight loss (kg) ranged across control groups from **-1.01 to 8.6 kg**	The mean weight loss (kg) in the intervention groups was **1.21 lower** (2.17 to 0.24 lower)
**Fasting blood glucose (mmol/l)** (IMPORTANT OUTCOME; measured with: Standard methods; Better indicated by lower values)											
128 (3 studies) 3 to 12 months	very serious[1,2,7,8]	serious[9]	no serious indirectness	no serious imprecision	undetected[5,10]	⊕⊖⊖⊖ **VERY LOW**[1,2,5,7,8,9,10] due to risk of bias, inconsistency	65	63	-	The mean fasting blood glucose (mmol/l) ranged across control groups from **-0.12 to -1.5 mmol/l**	The mean fasting blood glucose (mmol/l) in the intervention groups was **0.72 lower** (1.98 lower to 0.54 higher)
**HbA1c (%)** (IMPORTANT OUTCOME; measured with: High performance liquid chromatography (HPLC); Better indicated by lower values)											
227 (4 studies)	very serious[1,2,7,8]	no serious inconsistency	no serious indirectness	no serious imprecision	undetected[5,6]	⊕⊕⊖⊖ **LOW**[1,2,5,6,7,8] due to risk of bias	111	116	-	The mean hba1c (%) ranged across control	The mean hba1c (%) in the intervention groups was

3 to 12 months										groups from<br>**-0.19 to -1.1 %**	**0.1 lower**<br>(0.33 lower to 0.14 higher)
**High density lipoprotein (HDL) (mmol/l)** (IMPORTANT OUTCOME; measured with: Standard methods; Better indicated by lower values)											
226<br>(4 studies)<br>3 to 12 months	very serious[1,2,7,8]	no serious inconsistency	no serious indirectness	no serious imprecision	undetected[5,6]	⊕⊕⊖⊖<br>**LOW**[1,2,5,6,7,8]<br>due to risk of bias	110	116	**-**	The mean high density lipoprotein (hdl) (mmol/l) ranged across control groups from<br>**0 to 0.083 mmol/l**	The mean high density lipoprotein (hdl) (mmol/l) in the intervention groups was<br>**0.02 lower**<br>(0.07 lower to 0.04 higher)
**Low density lipoprotein (LDL) (mmol/l)** (IMPORTANT OUTCOME; measured with: Standard methods; Better indicated by lower values)											
226<br>(4 studies)<br>3 to 12 months	very serious[1,2,7,8]	no serious inconsistency	no serious indirectness	no serious imprecision	undetected[5,6]	⊕⊕⊖⊖<br>**LOW**[1,2,5,6,7,8]<br>due to risk of bias	110	116	**-**	The mean low density lipoprotein (ldl) (mmol/l) ranged across control groups from<br>**-0.05 to 0.0444 mmol/l**	The mean low density lipoprotein (ldl) (mmol/l) in the intervention groups was<br>**0.07 lower**<br>(0.25 lower to 0.1 higher)
**Systolic blood pressure (mmHg)** (IMPORTANT OUTCOME; measured with: Automated sphygmanometer; Better indicated by lower values)											
227<br>(4 studies)<br>3 to 12 months	very serious[1,2,7,8]	no serious inconsistency	no serious indirectness	no serious imprecision	undetected[5,6]	⊕⊕⊖⊖<br>**LOW**[1,2,5,6,7,8]<br>due to risk of bias	111	116	**-**	The mean systolic blood pressure (mmhg) ranged across control groups from<br>**-13 to 0.45 mmHg**	The mean systolic blood pressure (mmhg) in the intervention groups was<br>**4.41 lower**<br>(7.88 to 0.94 lower)
**Diastolic blood pressure** (IMPORTANT OUTCOME; measured with: Automated sphygmanometer; Better indicated by lower values)											
227<br>(4 studies)<br>3 to 12 months	very serious[1,2,7,8]	no serious inconsistency	no serious indirectness	no serious imprecision	undetected[5,6]	⊕⊕⊖⊖<br>**LOW**[1,2,5,6,7,8]<br>due to risk of bias	111	116	**-**	The mean diastolic blood pressure ranged across control groups from<br>**-7 to 4.23 mmHg**	The mean diastolic blood pressure in the intervention groups was<br>**3.61 lower**<br>(6.27 to 0.95 lower)

4.3 Summary of findings table – low protein diets in diabetic nephropathy

Low Protein Diet compared to Normal Protein Diet for Albuminuria in patients with type 2 diabetes

Patient or population: patients with Albuminuria in patients with type 2 diabetes
Settings:
Intervention: Low Protein Diet
Comparison: Normal Protein Diet

Outcomes	Illustrative comparative risks* (95% CI)		Relative effect (95% CI)	No of Participants (studies)	Quality of the evidence (GRADE)	Comments
	Assumed risk<br>Normal Protein Diet	Corresponding risk<br>Low Protein Diet				
**GFR**<br>Follow-up: 4 to 60 months	The mean gfr ranged across control groups from **-2.9 to -20.3 ml/min/1.73m²**	The mean gfr in the intervention groups was **4.65 higher** (0.32 lower to 9.62 higher)		478<br>(9 studies)	⊕⊕⊖⊖<br>**low**[1,2,3,4,5]	
**proteinuria**<br>Follow-up: 4 to 60 months	The mean proteinuria ranged across control groups from **210 to -233**	The mean proteinuria in the intervention groups was **0.4 standard deviations higher** (0.13 lower to 0.94 higher)		488<br>(11 studies)	⊕⊕⊖⊖<br>**low**[1,5,6,7,8]	SMD 0.4 (-0.13 to 0.94)

*The basis for the **assumed risk** (e.g. the median control group risk across studies) is provided in footnotes. The **corresponding risk** (and its 95% confidence interval) is based on the assumed risk in the comparison group and the **relative effect** of the intervention (and its 95% CI).

CI: Confidence interval;

GRADE Working Group grades of evidence
High quality: Further research is very unlikely to change our confidence in the estimate of effect.
Moderate quality: Further research is likely to have an important impact on our confidence in the estimate of effect and may change the estimate.
Low quality: Further research is very likely to have an important impact on our confidence in the estimate of effect and is likely to change the estimate.
Very low quality: We are very uncertain about the estimate.

[1] Most of the patients were not blinded to treatment allocation
[2] Four studies did not analyse all subjects in the groups which they were randomly allocated (intention-to-treat)
[3] Two studies had a high dropout rate (> 20 %)
[4] I^2 = 87% (Cochrane Handbook 5.0: considerable heterogeneity)
[5] Publication bias was not assessed as there were inadequate numbers of included trials to properly assess a funnel plot or more advanced regression-based assessments
[6] Five studies did not analyses all subjects in the group which they were randomly allocated (intention-to-treat)
[7] Three studies had a high dropout rate (> 20 %)
[8] I^2 =86% (Cochrane Handbook 5.0: considerable heterogeneity)

4.4 GRADE profile – low protein diets in diabetic nephropathy

Question: Low Protein Diet vs Normal Protein Diet for Albuminuria in patients with type 2 diabetes
Bibliography: Low Protein Diet for Albuminuria in patients with type 2 diabetes.

Quality assessment							Summary of Findings				
**Participants (studies) Follow up**	**Risk of bias**	**Inconsistency**	**Indirectness**	**Imprecision**	**Publication bias**	**Overall quality of evidence**	**Study event rates (%)**		**Relative effect** (95% CI)	**Anticipated absolute effects**	
							**With Normal Protein Diet**	**With Low Protein Diet**		**Risk with Normal Protein Diet**	**Risk difference with Low Protein Diet** (95% CI)
**GFR** (Better indicated by lower values)											
478 (9 studies) 4 to 60 months	serious[1,2,3]	serious[4]	no serious indirectness	no serious imprecision	undetected[5]	⊕⊕⊖⊖ **LOW**[1,2,3,4,5] due to risk of bias, inconsistency	245	233	-	The mean gfr ranged across control groups from **-2.9 to -20.3 ml/min/1.73m²**	The mean gfr in the intervention groups was **4.65 higher** (0.32 lower to 9.62 higher)
**proteinuria** (Better indicated by lower values)											
488 (11 studies) 4 to 60 months	serious[1,6,7]	serious[8]	no serious indirectness	no serious imprecision	undetected[5]	⊕⊕⊖⊖ **LOW**[1,5,6,7,8] due to risk of bias, inconsistency	245	243	-	The mean proteinuria ranged across control groups from **210 to -233**	The mean proteinuria in the intervention groups was **0.4 standard deviations higher** (0.13 lower to 0.94 higher)

5 Literature

1. Nezu U, K.H., Kondo Y, Sakuma M, Morimoto T, Ueda S, *Effect of low protein diet on kidney function in diabetic nephropathy metaanalyse of randomised controlled trials.* BMJ open, 2013.
2. Brinkworth, G.D., et al., *Long-term effects of advice to consume a high-protein, low-fat diet, rather than a conventional weight-loss diet, in obese adults with type 2 diabetes: one-year follow-up of a randomised trial.* Diabetologia, 2004. **47**(10): p. 1677-86.
3. Jesudason, D.R., E. Pedersen, and P.M. Clifton, *Weight-loss diets in people with type 2 diabetes and renal disease: a randomized controlled trial of the effect of different dietary protein amounts.* Am J Clin Nutr, 2013. **98**(2): p. 494-501.
4. Pedersen, E., D.R. Jesudason, and P.M. Clifton, *High protein weight loss diets in obese subjects with type 2 diabetes mellitus.* Nutr Metab Cardiovasc Dis, 2014. **24**(5): p. 554-62.
5. Larsen, R.N., et al., *The effect of high-protein, low-carbohydrate diets in the treatment of type 2 diabetes: a 12 month randomised controlled trial.* Diabetologia, 2011. **54**(4): p. 731-40.
6. Luger, M., et al., *Feasibility and efficacy of an isocaloric high-protein vs. standard diet on insulin requirement, body weight and metabolic parameters in patients with type 2 diabetes on insulin therapy.* Exp Clin Endocrinol Diabetes, 2013. **121**(5): p. 286-94.
7. Wycherley, T.P., et al., *A high-protein diet with resistance exercise training improves weight loss and body composition in overweight and obese patients with type 2 diabetes.* Diabetes Care, 2010. **33**(5): p. 969-76.
8. Krebs, J.D., et al., *The Diabetes Excess Weight Loss (DEWL) Trial: a randomised controlled trial of high-protein versus high-carbohydrate diets over 2 years in type 2 diabetes.* Diabetologia, 2012. **55**(4): p. 905-14.
9. Luscombe ND, C.P., Noakes M, Parker B, Wittert G, *Effects of energy-restricted diets containing increased protein on weight loss, resting energy expenditure, and the thermic effect of feeding in type 2 diabetes.* Diabetes Care, 2002. **25**(4): p. 652 - 657.
10. Parker B, L.N., Noakes M, Clifton P, *Effect of a high-protein, high-monounsaturated fat weight loss diet on glycemic control and lipid levels in type 2 diabetes.* Diabetes Care, 2002. **25**(3): p. 425 - 430.
11. von Bibra, H., et al., *Low-carbohydrate/high-protein diet improves diastolic cardiac function and the metabolic syndrome in overweight-obese patients with type 2 diabetes.* IJC Metabolic & Endocrine, 2014. **2**: p. 11-18.
12. Boden G, S.K., Homko C, Mozzoli M, Stein P, *Effect of a Low-Carbohydrate Diet on Appetite, Blood Glucose Levels, and Insulin Resistance in Obese Patients with Type 2 Diabetes.* American College of Physicians, 2005. **142**: p. 403 - 411.

13. Daly, M.E., et al., *Short-term effects of severe dietary carbohydrate-restriction advice in Type 2 diabetes--a randomized controlled trial.* Diabet Med, 2006. **23**(1): p. 15-20.
14. Davis, N.J., et al., *Differential effects of low-carbohydrate and low-fat diets on inflammation and endothelial function in diabetes.* J Diabetes Complications, 2011. **25**(6): p. 371-6.
15. Davis, N.J., et al., *Comparative study of the effects of a 1-year dietary intervention of a low-carbohydrate diet versus a low-fat diet on weight and glycemic control in type 2 diabetes.* Diabetes Care, 2009. **32**(7): p. 1147-52.
16. de Mello, V.D., et al., *A diet high in fatty fish, bilberries and wholegrain products improves markers of endothelial function and inflammation in individuals with impaired glucose metabolism in a randomised controlled trial: the Sysdimet study.* Diabetologia, 2011. **54**(11): p. 2755-67.
17. Dyson, P.A., *A review of low and reduced carbohydrate diets and weight loss in type 2 diabetes.* J Hum Nutr Diet, 2008. **21**(6): p. 530-8.
18. Dyson, P.A., S. Beatty, and D.R. Matthews, *A low-carbohydrate diet is more effective in reducing body weight than healthy eating in both diabetic and non-diabetic subjects.* Diabet Med, 2007. **24**(12): p. 1430-5.
19. Elhayany, A., et al., *A low carbohydrate Mediterranean diet improves cardiovascular risk factors and diabetes control among overweight patients with type 2 diabetes mellitus: a 1-year prospective randomized intervention study.* Diabetes Obes Metab, 2010. **12**(3): p. 204-9.
20. Nuttall, F.Q. and M.C. Gannon, *Metabolic response of people with type 2 diabetes to a high protein diet.* Nutr Metab (Lond), 2004. **1**(1): p. 6.
21. Gutierrez, M., et al., *Utility of a short-term 25% carbohydrate diet on improving glycemic control in type 2 diabetes mellitus.* J Am Coll Nutr, 1998. **17**(6): p. 595-600.
22. Hussain, T.A., et al., *Effect of low-calorie versus low-carbohydrate ketogenic diet in type 2 diabetes.* Nutrition, 2012. **28**(10): p. 1016-21.
23. Keogh, J.B., et al., *Long-term weight maintenance and cardiovascular risk factors are not different following weight loss on carbohydrate-restricted diets high in either monounsaturated fat or protein in obese hyperinsulinaemic men and women.* Br J Nutr, 2007. **97**(2): p. 405-10.
24. Kirk, J.K., et al., *Restricted-carbohydrate diets in patients with type 2 diabetes: a meta-analysis.* J Am Diet Assoc, 2008. **108**(1): p. 91-100.
25. Khoo, J., et al., *Comparing effects of a low-energy diet and a high-protein low-fat diet on sexual and endothelial function, urinary tract symptoms, and inflammation in obese diabetic men.* J Sex Med, 2011. **8**(10): p. 2868-75.

26. McAuley, K.A., et al., *Long-term effects of popular dietary approaches on weight loss and features of insulin resistance.* Int J Obes (Lond), 2006. **30**(2): p. 342-9.
27. McAuley, K.A., et al., *Comparison of high-fat and high-protein diets with a high-carbohydrate diet in insulin-resistant obese women.* Diabetologia, 2005. **48**(1): p. 8-16.
28. McCarthy, E.M. and M.E. Rinella, *The role of diet and nutrient composition in nonalcoholic Fatty liver disease.* J Acad Nutr Diet, 2012. **112**(3): p. 401-9.
29. Navas-Carretero, S., et al., *Chronologically scheduled snacking with high-protein products within the habitual diet in type-2 diabetes patients leads to a fat mass loss: a longitudinal study.* Nutr J, 2011. **10**: p. 74.
30. Papakonstantinou, E., et al., *A high-protein low-fat diet is more effective in improving blood pressure and triglycerides in calorie-restricted obese individuals with newly diagnosed type 2 diabetes.* Eur J Clin Nutr, 2010. **64**(6): p. 595-602.
31. Samaha, F.F., et al., *A low-carbohydrate as compared with a low-fat diet in severe obesity.* N Engl J Med, 2003. **348**(21): p. 2074-81.
32. Seshadri, P., et al., *A randomized study comparing the effects of a low-carbohydrate diet and a conventional diet on lipoprotein subfractions and C-reactive protein levels in patients with severe obesity.* Am J Med, 2004. **117**(6): p. 398-405.
33. Stern, L., et al., *The effects of low-carbohydrate versus conventional weight loss diets in severely obese adults: one-year follow-up of a randomized trial.* Ann Intern Med, 2004. **140**(10): p. 778-85.
34. Tay, J., et al., *A Very Low-Carbohydrate, Low-Saturated Fat Diet for Type 2 Diabetes Management: A Randomized Trial.* Diabetes Care, 2014. **37**(11): p. 2909-2918.
35. Westman, E.C., et al., *The effect of a low-carbohydrate, ketogenic diet versus a low-glycemic index diet on glycemic control in type 2 diabetes mellitus.* Nutr Metab (Lond), 2008. **5**: p. 36.
36. Wheeler, M.L., et al., *Macronutrients, food groups, and eating patterns in the management of diabetes: a systematic review of the literature, 2010.* Diabetes Care, 2012. **35**(2): p. 434-45.
37. Wolever, T.M., et al., *The Canadian Trial of Carbohydrates in Diabetes (CCD), a 1-y controlled trial of low-glycemic-index dietary carbohydrate in type 2 diabetes: no effect on glycated hemoglobin but reduction in C-reactive protein.* Am J Clin Nutr, 2008. **87**(1): p. 114-25.
38. Yancy, W.S., Jr., et al., *A randomized trial of a low-carbohydrate diet vs orlistat plus a low-fat diet for weight loss.* Arch Intern Med, 2010. **170**(2): p. 136-45.
39. Azadbakht, L., S. Atabak, and A. Esmaillzadeh, *Soy protein intake, cardiorenal indices, and C-reactive protein in type 2 diabetes with nephropathy: a longitudinal randomized clinical trial.* Diabetes Care, 2008. **31**(4): p. 648-54.

40. Pecis, M., M.J. de Azevedo, and J.L. Gross, *Chicken and fish diet reduces glomerular hyperfiltration in IDDM patients.* Diabetes Care, 1994. **17**(7): p. 665-72.
41. Wheeler, M.L., et al., *Animal versus plant protein meals in individuals with type 2 diabetes and microalbuminuria: effects on renal, glycemic, and lipid parameters.* Diabetes Care, 2002. **25**(8): p. 1277-82.
42. Gross, J.L., et al., *Effect of a chicken-based diet on renal function and lipid profile in patients with type 2 diabetes: a randomized crossover trial.* Diabetes Care, 2002. **25**(4): p. 645-51.
43. Pipe, E.A., et al., *Soy protein reduces serum LDL cholesterol and the LDL cholesterol:HDL cholesterol and apolipoprotein B:apolipoprotein A-I ratios in adults with type 2 diabetes.* J Nutr, 2009. **139**(9): p. 1700-6.
44. Dullaart RPF, B.B., Meijer S, Van Doormaal JJ, Sluiter WJ, *Long-term effects of protein-restricted diet on albuminuria.* Diabetes Care, 1993. **16**(2): p. 483 - 492.
45. Dussol, B., et al., *A randomized trial of low-protein diet in type 1 and in type 2 diabetes mellitus patients with incipient and overt nephropathy.* J Ren Nutr, 2005. **15**(4): p. 398-406.
46. Hansen HP, T.-L.E., Jensen BR, Parving HH, *Effect of Dietary Protein Restriction on Prognosis in patients with diabetic nephropathy.* Kidney Int, 2002. **62**: p. 220 - 228.
47. Koya, D., et al., *Long-term effect of modification of dietary protein intake on the progression of diabetic nephropathy: a randomised controlled trial.* Diabetologia, 2009. **52**(10): p. 2037-45.
48. Meloni, C., et al., *Severe dietary protein restriction in overt diabetic nephropathy: benefits or risks?* J Ren Nutr, 2002. **12**(2): p. 96-101.
49. Pijls LTJ, D.V.H., Van Eijk JThM, Donker AJM, *Protein restriction, glomerular filtration rate and albuminuria in patients with type 2 diabetes mellitus: a randomised trial.* European Journal of Clinical Nutrition, 2002. **56**: p. 1200 - 1207.
50. Raal FJ, K.W., Lawson M, Esser JD, Buys R, Fourie L, Panz VR, *Effect of moderate dietary protein restriction on the progression of overt diabetic nephropathy: a 6 mo prospective study.* Am J Clin Nutr, 1994. **60**: p. 579 - 85.
51. Velazquez Lopez L, A.J., Goycochea Robles MV, Tamayo MT, Limones RC, *Effect of protein restriction diet on renal function and metabolic control in patients with type 2 diabetes: a randomised controlled trial.* Nutricion Hospitalaria, 2008. **23**: p. 141 - 147.
52. Walker JD, D.R., Murrells TJ, Bending JJ, Mattock MB, Keen H, Viberti GC, *Restriction of Dietary Protein and Progression of Renal Failure in Diabetic Nephropathy.* The Lancet, 1989: p. 1411 - 1414.
53. Zeller K, W.E., Sullivan L, Raskin P, Jacobson HR, *Effect of Restricting Dietary Protein on the progression of renal failure in patients with insulin-dependent diabetes mellitus.* The new England Journal of Medicine, 1991. **324**: p. 78 - 84.

54. Kasiske, B.L., et al., *A meta-analysis of the effects of dietary protein restriction on the rate of decline in renal function.* Am J Kidney Dis, 1998. **31**(6): p. 954-61.
55. Maeda Y, S.T., *Diet therapy in diabetic nephropathy.* Nutrition and Kidney Disease: A new Era, 2007. **155**: p. 50 - 58.
56. Otoda, T., K. Kanasaki, and D. Koya, *Low-protein diet for diabetic nephropathy.* Curr Diab Rep, 2014. **14**(9): p. 523.
57. Pan Y, G.L., Jin HM, *Low protein diet for diabetic nephropathy Meta-analysis.* Am J Clin Nutr, 2008. **88**: p. 660 - 666.
58. Pedrini MT, L.A., Lau J, Chalmers TC, Wang PH, *The effect of dietary protein restriction on the progression of renal disease.* Ann Intern Med, 1996. **126**: p. 627 - 632.
59. Robertson LM, W.N., Robertson A, *Protein restriction for diabetic renal disease* in *The cochrane library*. 2007.
